# The Role of ExoS in Dissemination of *Pseudomonas aeruginosa* during Pneumonia

**DOI:** 10.1371/journal.ppat.1004945

**Published:** 2015-06-19

**Authors:** Stephanie M. Rangel, Maureen H. Diaz, Claire A. Knoten, Angelica Zhang, Alan R. Hauser

**Affiliations:** 1 Departments of Microbiology/Immunology, Feinberg School of Medicine, Northwestern Univesity, Chicago, Illinois, United States of America; 2 Department of Medicine, Feinberg School of Medicine, Northwestern University, Chicago, Illinois, United States of America; University of Washington, UNITED STATES

## Abstract

Hospital-acquired pneumonia is associated with high rates of morbidity and mortality, and dissemination to the bloodstream is a recognized risk factor for particularly poor outcomes. Yet the mechanism by which bacteria in the lungs gain access to the bloodstream remains poorly understood. In this study, we used a mouse model of *Pseudomonas aeruginosa* pneumonia to examine this mechanism. *P*. *aeruginosa* uses a type III secretion system to deliver effector proteins such as ExoS directly into the cytosol of eukaryotic cells. ExoS, a bi-functional GTPase activating protein (GAP) and ADP-ribosyltransferase (ADPRT), inhibits phagocytosis during pneumonia but has also been linked to a higher incidence of dissemination to the bloodstream. We used a novel imaging methodology to identify ExoS intoxicated cells during pneumonia and found that ExoS is injected into not only leukocytes but also epithelial cells. Phagocytic cells, primarily neutrophils, were targeted for injection with ExoS early during infection, but type I pneumocytes became increasingly injected at later time points. Interestingly, injection of these pneumocytes did not occur randomly but rather in discrete regions, which we designate ““**f**ields **o**f **c**ell **i**njection” (FOCI). These FOCI increased in size as the infection progressed and contained dead type I pneumocytes. Both of these phenotypes were attenuated in infections caused by bacteria secreting ADPRT-deficient ExoS, indicating that FOCI growth and type I pneumocyte death were dependent on the ADPRT activity of ExoS. During the course of infection, increased FOCI size was associated with enhanced disruption of the pulmonary-vascular barrier and increased bacterial dissemination into the blood, both of which were also dependent on the ADPRT activity of ExoS. We conclude that the ADPRT activity of ExoS acts upon type I pneumocytes to disrupt the pulmonary-vascular barrier during *P*. *aeruginosa* pneumonia, leading to bacterial dissemination.

## Author Summary

Dissemination to the bloodstream is a poor prognostic sign in patients with hospital-acquired pneumonia, yet the mechanism by which this occurs is poorly understood. To begin to address this issue, we have used a mouse model of *P*. *aeruginosa* pneumonia to study the mechanism by which the type-III-secreted effector protein ExoS enhances bacterial dissemination. We show that intoxication of type I pneumocytes by ExoS leads to cell death and disruption of the pulmonary-vascular barrier, allowing bacterial dissemination into the bloodstream. These effects required the ADP-ribosyltransferase activity of ExoS, as strains secreting an ExoS variant lacking this activity demonstrated reduced type I pneumocytes death and pulmonary-vascular breakdown. This study indicates that inhibitors of the ADP-ribosyltransferase activity of ExoS could serve as novel therapeutics for the prevention of bacteremic pneumonia.

## Introduction

Hospital-acquired pneumonia (HAP) is a severe form of nosocomial infection associated with attributable mortality rates of approximately 30% [1]. Dissemination of bacteria from the lung to the bloodstream is a particularly poor prognostic sign in HAP [2]. Yet the mechanisms by which bacteria disrupt the pulmonary-vascular barrier to reach the bloodstream remain largely unexplored. *P*. *aeruginosa* is the cause of approximately 15–20% of HAP cases [3–5], and *P*. *aeruginosa* bacteremic pneumonia is associated with mortality rates considerably higher than non-bacteremic pneumonia [6,7]. Among the many virulence determinants of *P*. *aeruginosa* is a type III secretion system that injects toxic effector proteins directly into the cytosol of host cells [8]. *P*. *aeruginosa* secretes four known effector proteins by this pathway: ExoS, ExoT, ExoU, and ExoY. A functional type III secretion system has been associated with worse clinical outcomes and higher mortality rates in patients with *P*. *aeruginosa* pneumonia [9,10].

Approximately 70% of clinical strains contain the gene encoding ExoS [11], underscoring the importance of understanding its contribution to *P*. *aeruginosa* pathogenesis. ExoS is a bi-functional effector protein, with GTPase activating protein (GAP) and ADP-ribosyltransferase (ADPRT) activities. The GAP and ADPRT domains of ExoS have been implicated in cell rounding, apoptosis, bleb-niche formation, and anti-internalization phenotypes using in vitro assays [12–16]. Likewise, ExoS secretion has been associated with more severe disease in several animal models, including a mouse model of pneumonia [17,18]. In both pneumonia and burn models, secretion of ExoS led to higher rates of dissemination from the site of infection, which may have contributed to the worse outcomes [18,19]. However, the mechanism by which ExoS causes dissemination remains unclear.

There are few studies examining the effects of ExoS on pulmonary alveolar epithelial cells due in part to difficulties in isolating and culturing these cell types from mouse lungs. Lung alveoli consist of two types of epithelial cells, type I pneumocytes and type II pneumocytes, both of which are important for defense against bacterial pathogens. Type I pneumocytes are flat, elongated cells that are responsible for gas exchange within the lungs [20]. Along with the endothelial cells in lung capillaries and the basement membrane components, type I pneumocytes comprise the pulmonary-vascular barrier that prevents bacterial pathogens from entering the blood. Type II pneumocytes are smaller cuboidal cells that produce and secrete surfactant [20]. Surfactant decreases surface tension to facilitate lung expansion, interacts with resident macrophages to enhance phagocytosis of pathogens, and creates a physical barrier for entrapping bacteria [21]. Lung epithelial cells are also a major source of cytokines and chemokines that mediate rapid recruitment of immune cells into the lungs during infection. We hypothesized that interactions between ExoS and alveolar epithelial cells might contribute to the ability of *P*. *aeruginosa* to disseminate from the lungs to the bloodstream during pneumonia.

In the present study, we used a novel in situ approach to examine the role of ExoS in acute pneumonia. We showed that early during pneumonia only phagocytic cells were appreciably injected with ExoS. However at later time points, clusters of type I pneumocytes were injected, and these discrete foci of injected cells increased in size as the infection progressed. The growth of these foci was associated with increased disruption of the pulmonary-vascular barrier and with increased bacterial dissemination into the bloodstream. We conclude that type I pneumocyte intoxication with ExoS causes breakdown of the pulmonary-vascular barrier, allowing bacterial dissemination during pneumonia.

## Results

### ExoS is injected into recruited neutrophils early during pneumonia

**Fig 1:**
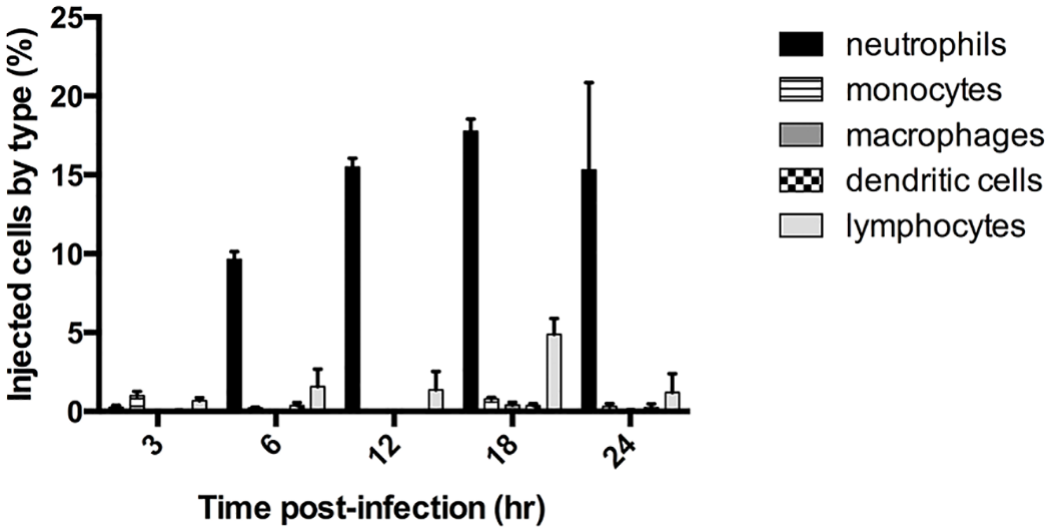
ExoS is injected into neutrophils during early pneumonia. Mice were infected with PA99Sbla, a *P*. *aeruginosa* strain secreting an ExoS-β-lactamase fusion protein. Leukocytes were recovered and analyzed by flow cytometry after staining with CCF2-AM and cell discriminatory markers. Lymphocyte numbers represent a combination of T helper cells, cytotoxic T cells, B cells, and NK cells. The data represent the means ± standard errors of the mean (SEM). n≥ 3 mice per time point per experiment; the experiment was repeated three times; one representative result is shown.

We previously showed that ExoS caused a heightened inflammatory response in the lungs, with the majority of infiltrating cells being neutrophils [22]. Furthermore, ExoS inhibited phagocytosis by these neutrophils [18,22]. Despite the ability of *P*. *aeruginosa* to inject a variety of cell types in vitro, we hypothesized that phagocytic cells would comprise the majority of intoxicated cells in vivo during early infection. We analyzed ExoS leukocyte injection using a mouse model of pneumonia and a β-lactamase reporter assay. In this assay, a fluorogenic β-lactam substrate, CCF2-AM, is applied to infected cells to detect translocation of ExoS fused with a β-lactam tag [23,24]. After diffusion into host cells, intact CCF2-AM exhibits green fluorescence. However, in the presence of injected ExoS containing a β-lactamase tag, CCF2-AM is cleaved, resulting in blue fluorescence. Cells can then be categorized as injected or not injected based upon their blue:green fluorescence ratio. Infections were performed using a variant of the clinical isolate PA99, which naturally secretes ExoU, ExoT, and ExoS [18]. We wished to study the contribution of ExoS to disease progression in the absence of confounding effects caused by ExoU and ExoT. To accomplish this, we used a previously generated strain of PA99 with disruptions in *exoU*, *exoT*, and *exoS* (designated PA99null [18]). PA99null was complemented at a neutral chromosomal site with a single copy of an *exoS* allele encoding ExoS with a C-terminal β-lactamase tag. This *exoS* allele is under the control of its endogenous promoter. We designated this strain PA99Sbla. We infected mice with PA99Sbla and recovered leukocytes from minced whole lungs at various time points post-infection to analyze the injected cell types. As expected, neutrophils were the predominant white blood cell type injected with ExoS (Fig 1). A substantial proportion of neutrophils were injected as early as 6 hr post-infection, and this proportion increased as the infection progressed. By 24 hr post-infection, approximately 15% of recovered neutrophils were injected with ExoS. It is plausible that this is an underestimation of the actual number of neutrophils injected with ExoS since at least 100 β-lactamase molecules are required per cell for detection of injection [23]. These results demonstrate that neutrophils are the leukocyte type most frequently injected with ExoS during early pneumonia.

### ExoS is injected into type I pneumocytes at later time points during infection

While abundant numbers of leukocytes can be recovered from minced whole lungs, epithelial cells such as type I and type II pneumocytes remain entrapped in large aggregates and cannot be analyzed. To determine whether ExoS intoxication of these cells might also occur, we adapted the CCF2-AM/ β-lactamase reporter assay for use on lung tissue sections. Unlike previous studies, this in situ analysis allowed us to study toxin injection in the context of intact lung tissue architecture [25,26]. For these experiments, whole lungs were excised from mice with pneumonia and incubated with CCF2-AM while intact to allow for β-lactamase cleavage of the fluorogenic substrate. The lungs were then fixed, frozen, and sectioned, and individual sections were stained with cell specific markers. We then used the TissueFAXS system (see Materials and Methods) to image cross-sections of entire lobes of the lungs (S1A Fig). Next, TissueQuest software was used to identify and count injected cells based upon their blue:green fluorescence ratio (S1B Fig). Blue:green fluorescence thresholds for detection of injected cells were determined by first using lung sections from mice infected with a strain secreting ExoS lacking a β-lactamase tag to set background fluorescence levels. Type I pneumocytes, type II pneumocytes, and phagocytic cells (neutrophils and monocytes) were identified and counted based upon staining with antibodies recognizing caveolin-1, pSP-C, or Gr1, respectively (S1C–S1E Fig). A detailed description of the algorithm used to identify injected cells of each cell type is provided in S2 Fig and the Materials and Methods section.

**Fig 2:**
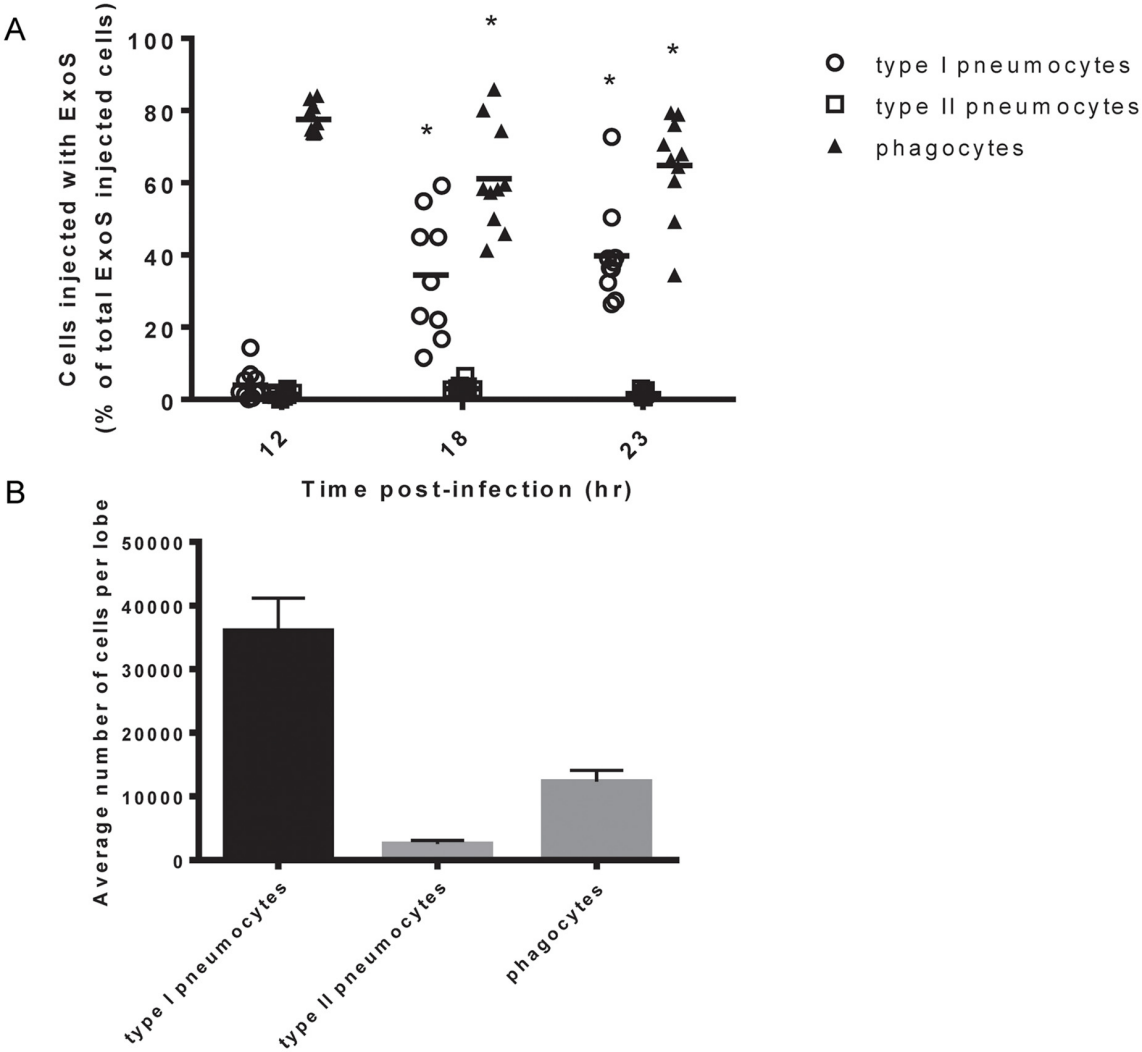
Type I pneumocytes comprise an increasing proportion of injected cells over time during pneumonia. Mice were infected with PA99Sbla, and lung tissue sections across an entire lung lobe were analyzed. A) The proportion of total injected cells that were type I pneumocytes, type II pneumocytes, or phagocytes was measured over time. Cell types were defined as follows: phagocytes, Gr1^+^ cells; type I pneumocytes, caveolin-1^+^ cells; and type II pneumocytes, pSP-C^+^ cells. Each data point represents a tissue section. One tissue section was analyzed per lung lobe, with two lobes analyzed per mouse and at least 4 mice per condition, for a total of at least 8 tissue sections per condition. Bars indicate medians. *, significantly different from 12 hr time point. B) The total number of type I pneumocytes, type II pneumocytes, and phagocytic cells within lung sections was quantified using the TissueQuest software. Values represent the mean number of cells per lobe cross-section based on the analysis of 10 lobes from 5 mice following 23 hr of infection with PA99Sbla. Error bars represent SEM.

Mice were infected with PA99Sbla and sacrificed at various times post-infection. Lungs were removed and analyzed for injected cells. At 12 hr post-infection, phagocytes were the most frequently injected cell type within lung tissue sections (Fig 2A), confirming our flow cytometry injection results (Fig 1). Phagocytes continued to comprise a large proportion of injected cells at 18 hr and 23 hr post-infection. Interestingly, relatively few type I pneumocytes were injected at 12 hr post-infection, but this cell type became increasingly injected at 18 and 23 hr post-infection (Fig 2A). In contrast, type II pneumocytes represented a very small proportion of the total ExoS-injected cells even 23 hr after infection. This may be because type II pneumocytes are of relatively low abundance within the lungs (Fig 2B) or because they less frequently make contact with *P*. *aeruginosa* bacteria relative to type I pneumocytes and phagocytic cells. Alternatively, *P*. *aeruginosa* type III secretion may preferentially inject phagocytic cells and type I pneumocytes relative to type II pneumocytes. These results indicate that phagocytes are injected early during the course of *P*. *aeruginosa* pneumonia, that type I pneumocytes become increasingly injected at later time points, and that type II pneumocytes are not appreciably injected even at 23 hr after infection.

### Focal areas of ExoS-injected type I pneumocytes appear and increase in size during pneumonia

**Fig 3:**
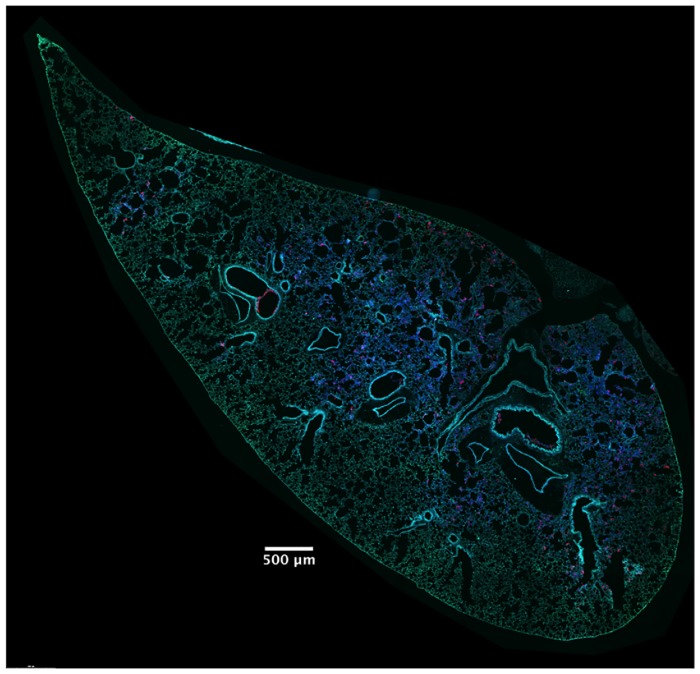
High concentrations of ExoS-injected cells occur in discrete regions within the lungs. A representative lung section from a mouse infected for 18 hr. ExoS-injected cells are blue and uninjected cells are green. Contrast and brightness for all color filters were uniformly adjusted over the entire image for better visualization.

**Fig 4:**
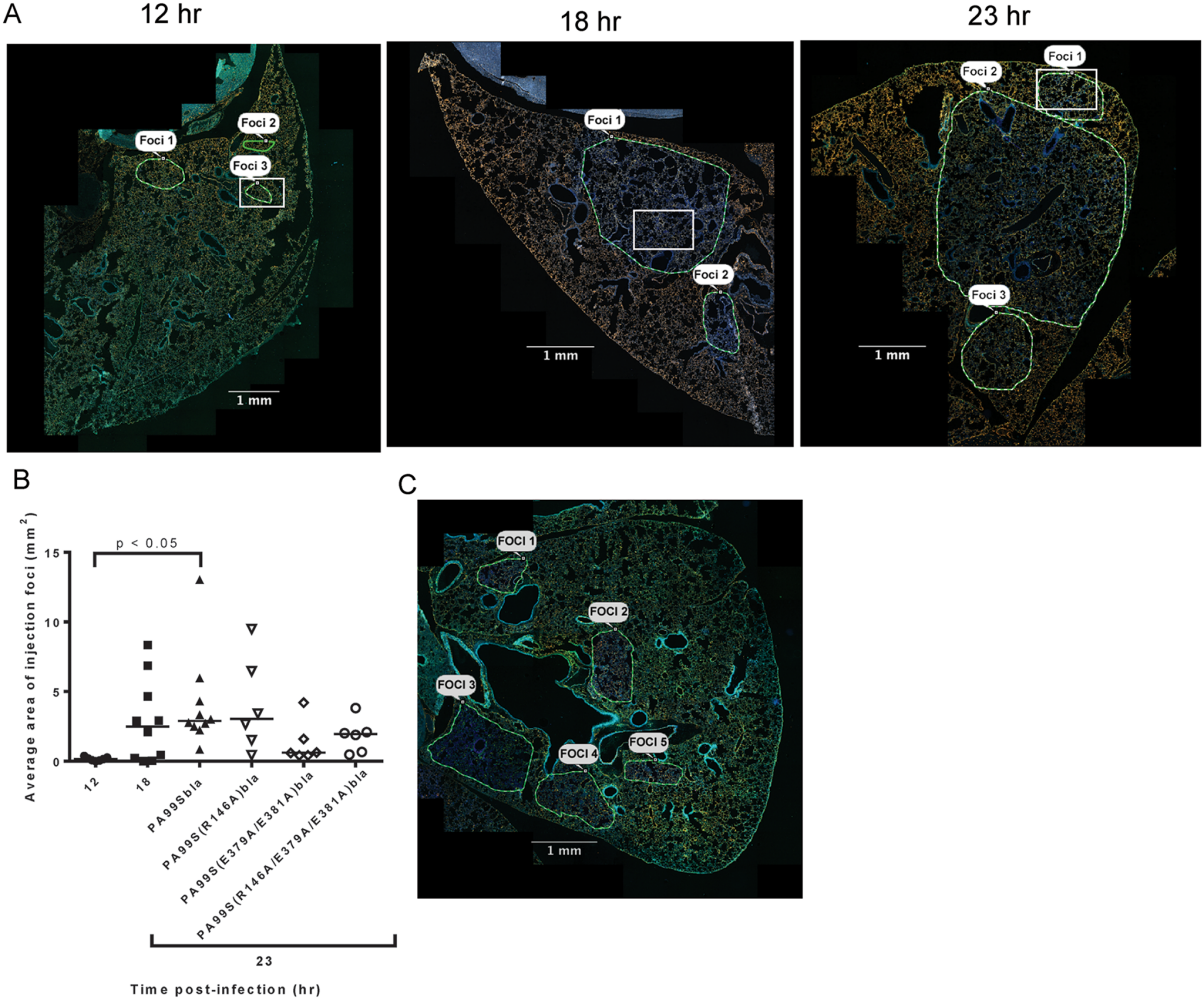
Regions with high densities of injected cells increase in size with duration of infection. A) Representative images of lung sections demonstrating regions with high concentrations of ExoS-injected cells. These regions were outlined by visual examination. Magnified images from within the regions outlined by white boxes are shown in S4 Fig. B) The areas of the outlined regions were measured using the TissueQuest software. Each data point represents the average area of all measured regions of injection across one lobe of a mouse infected with PA99Sbla for 12, 18, or 23 hr, or with strains secreting the enzymatically inactive ExoS variants for 23 hr. Bars indicate medians. For PA99Sbla-infected mice, the 12 hr and 23 hr time points were statistically different. For the 23 hr time point values, the overall ANOVA *P* = 0.05, but individual pairwise comparisons were not statistically significant. C) Image of a representative lung lobe cross-section from a mouse infected with PA99S(E379A/E381A)bla (deficient in ADPRT activity) at the 23 hr time point showing smaller, more numerous FOCI. In panels A and C, the contrast and brightness for all color filters were uniformly adjusted over the entire image for better visualization. Scale bars represent 1 mm.

In addition to identifying and counting injected cells, the TissueFAXS imaging system has the capability of marking these cells within the tissue sections of entire lung lobes. In this manner, we were able to examine the spatial distribution of ExoS-injected cells (S3A Fig). We noticed that injected cells were not uniformly distributed throughout the lung sections but rather often occurred in distinct regions (Fig 3 and S3B–S3D Fig). Examination of lungs infected for 12, 18, and 23 hr indicated that these regions of injected cells increased in size as the pneumonia progressed (Fig 4A and 4B). Furthermore, the concentration of injected cells within these regions increased over time (S4 Fig). Injection appeared to start at a few distinct sites within the lungs. These regions of injection subsequently expanded outward and coalesced as the infection progressed. We will refer to these regions of injected cells as “**f**ields **o**f **c**ell **i**njection” (FOCI).

**Fig 5:**
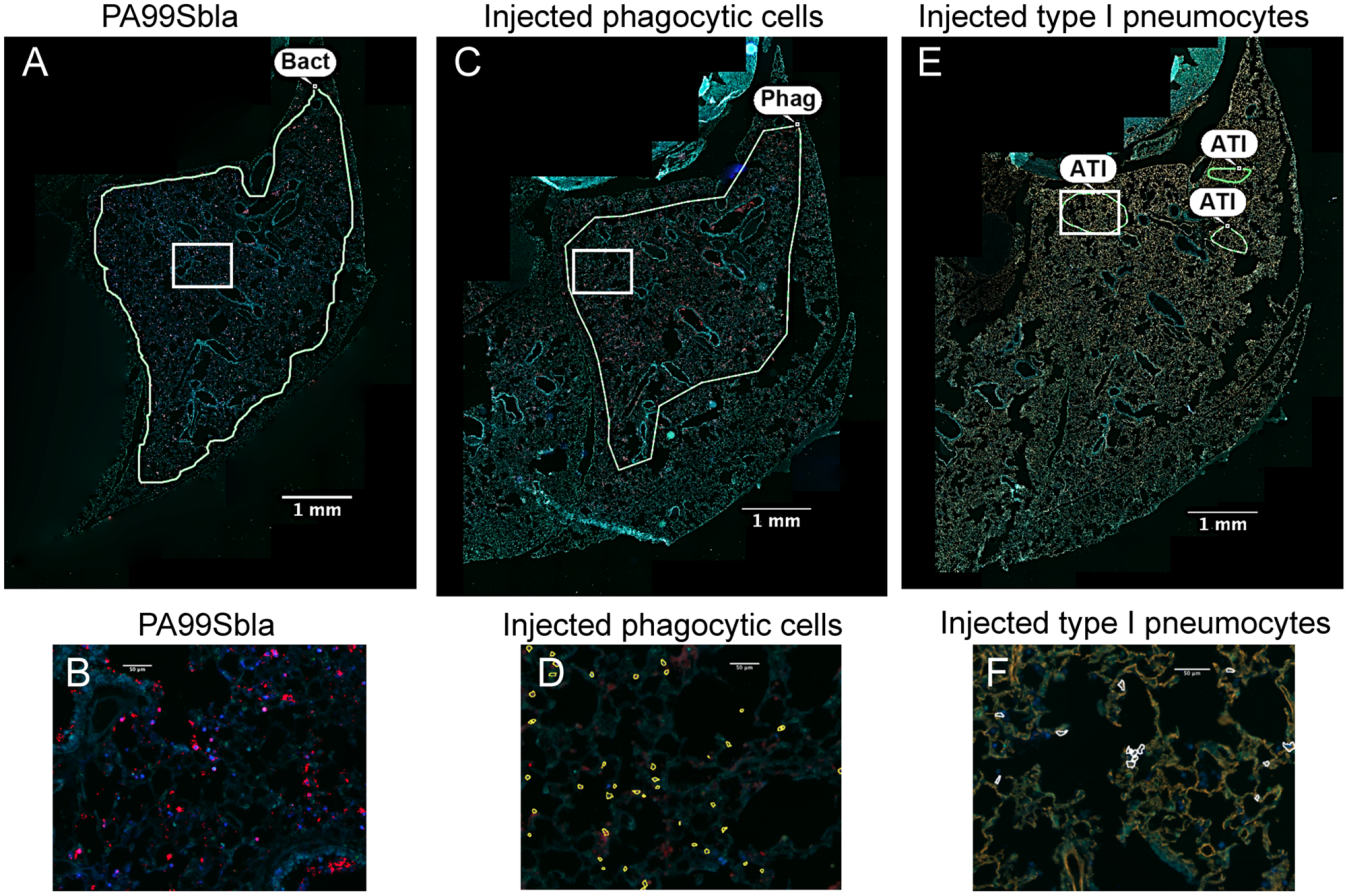
The distribution of injected type I pneumocytes differs from that of phagocytes or bacteria within lung lobes. Tissue sections of lung lobes obtained at 12 hr post-infection were analyzed for bacteria (PA99Sbla) (A-B), injected phagocytic cells (C-D), and injected type I pneumocytes (E-F). White boxes in panels A, C, and E are depicted at higher magnification in panels B, D, and F, respectively. In panels A & B bacteria are stained red. In panels C & D, phagocytes are stained red. In panels E & F, type I pneumocytes are stained orange. In panels D and F, injected cells were identified using the TissueQuest software and marked with outlines. Yellow outlines in panel D are injected phagocytes; white outlines in panel F are injected type I pneumocytes. Manually drawn outlines were added to panels A, C, and E to designate regions containing appreciable numbers of the indicated cell types. Bacteria and injected phagocytic cells were widely dispersed throughout the lobe (A, C). In contrast, injection of type I pneumocytes mostly occurred in small discrete regions within the larger regions containing bacteria and injected phagocytes. In panels A, C and E, the contrast and brightness for all color filters were uniformly adjusted over the entire image for better visualization. Bact, bacteria; Phag, phagocytes; ATI, type I pneumocytes.

We next examined the cell types that were injected within FOCI. We first examined lung sections taken from mice at a relatively early stage of infection (12 hr), when small FOCI were just becoming apparent. Even at this early time point, injected phagocytes were present throughout large regions of the lungs (Fig 5C and 5D). These injected phagocytes caused a diffuse background of light blue throughout much of the lung. As expected, these regions corresponded to the distribution of bacteria in the lungs (Fig 5A and 5B). Within the diffuse background of injected phagocytes, small FOCI were apparent as areas of more intense blue fluorescence. These FOCI contained injected type I pneumocytes (Fig 5E and 5F and S4A Fig), as well as numerous bacteria (S5 Fig). At later time points, more and more of the type I pneumocytes within FOCI became injected, and the regions of injected type I pneumocytes increased in size (S4 Fig). Together with our time-course results (Fig 2A), these observations indicate that bacteria were widely distributed in the lungs by 12 hr post-infection, and that these bacteria were competent to inject phagocytes. Within this background of injected phagocytes, small FOCI consisting of injected type I pneumocytes formed and subsequently increased in size and intensity.

### FOCI formation also occurs during infection with other *P*. *aeruginosa* strains

**Fig 6:**
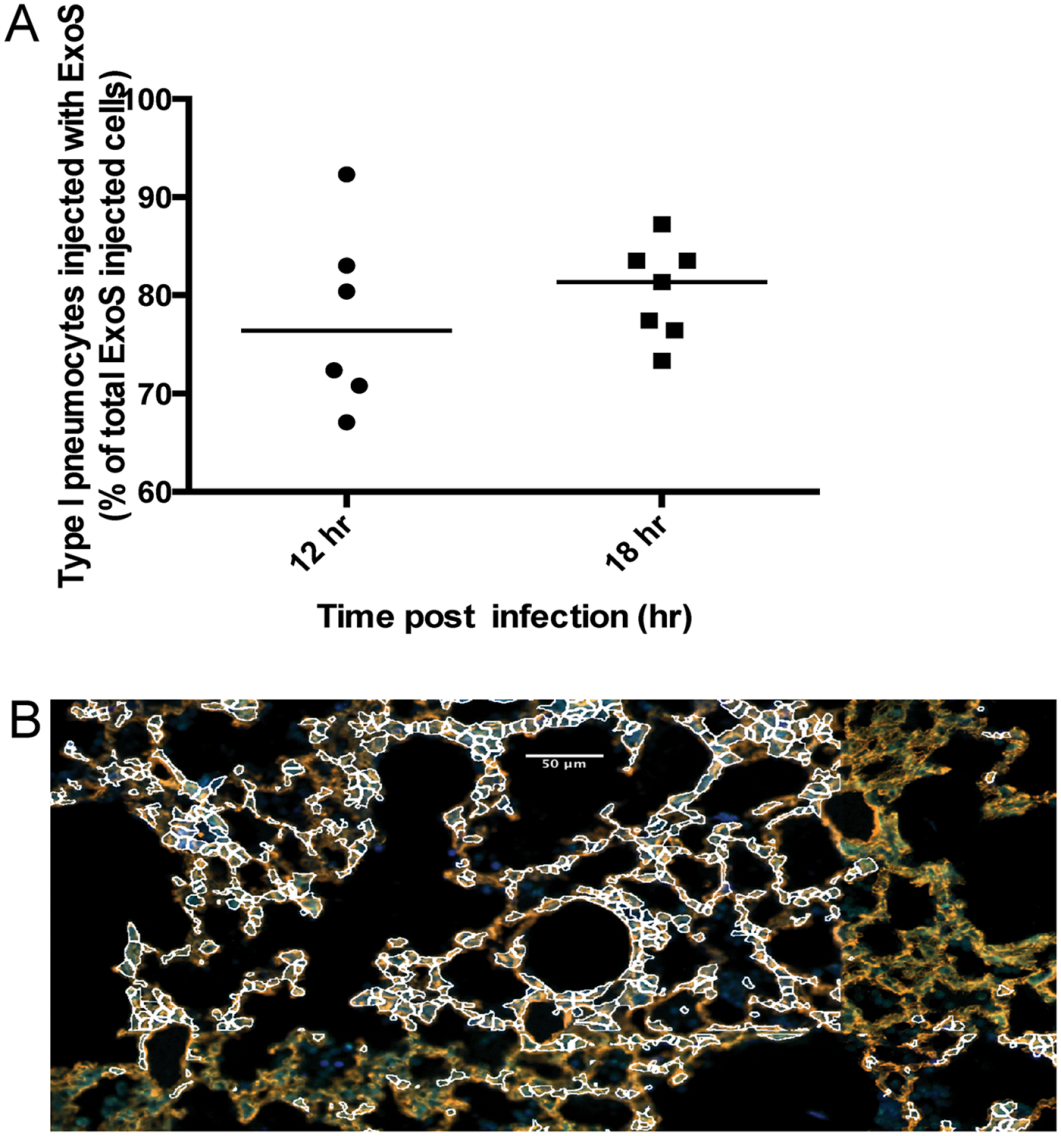
A clinical *P*. *aeruginosa* isolates that naturally secretes both ExoS and ExoT also causes formation of FOCI. Mice were infected with BL12+ S(R146A/E379A/E381A)bla and sacrificed at 12 and 18 hr post-infection. (Mice did not survive to 23 hr, preventing assessments at this later time point.) A) Proportion of total injected cells that were type I pneumocytes. Lung tissue sections encompassing an entire lung lobe were imaged to evaluate the localization of injected type I pneumocytes. Each data point represents a tissue section. One tissue section was analyzed per lung lobe, with at least two lobes analyzed per mouse and 3 mice per condition, for a total of at least 6 tissue sections per condition. Bars indicate medians. B) Representative image of a FOCI at 12 hr. Scale bar represents 50 μm.

**Fig 7:**
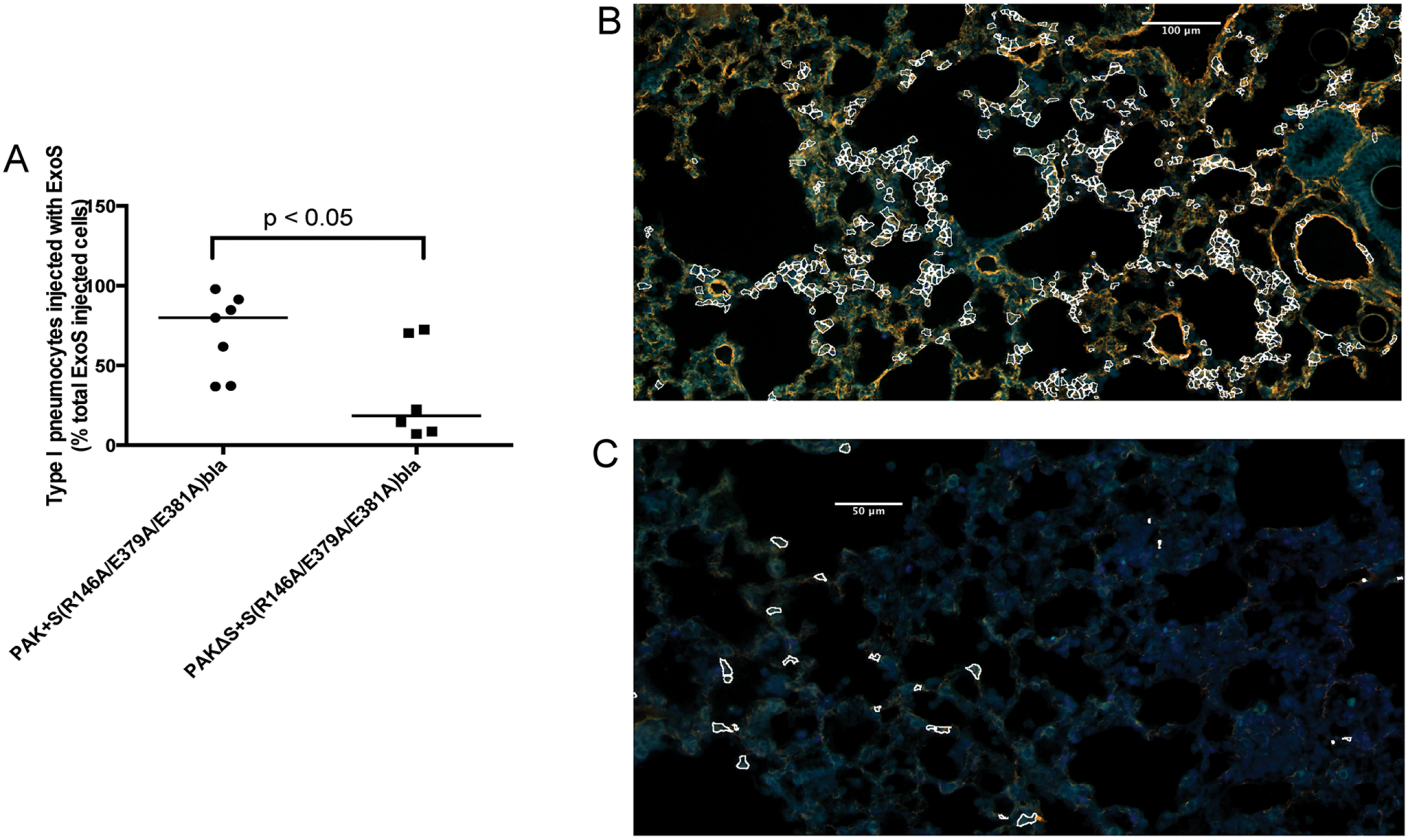
Secretion of ExoT was not sufficient to cause FOCI formation. Mice were infected with PAK+S(R146A/E379A/E381A)bla or PAKΔS+S(R146A/E379A/E381A)bla. At 23 hr post-infection, mice were sacrificed, and their lungs were removed, stained, and sectioned to determine the localization of injected type I pneumocytes. A) Type I pneumocyte injection by PAK+S(R146A/E379A/E381A)bla (ExoS^+^, ExoT^+^) compared to PAKΔS+S(R146A/E379A/E381A)bla (ExoT^+^). Each data point represents a tissue section. One tissue section was analyzed per lung lobe, with at least two lobes analyzed per mouse and 3 mice per condition, for a total of at least 6 tissue sections per condition. Bars indicate medians. B) Representative image of a FOCI from a mouse infected with PAK+S(R146A/E379A/E381A)bla (ExoS^+^, ExoT^+^). Injected type I pneumocytes are outlined in white. Scale bar represents 100 μm. C) Representative image of a FOCI from a mouse infected with PAKΔS+S(R146A/E379A/E381A)bla (ExoT^+^). Injected type I pneumocytes are outlined in white. Scale bar represents 50 μm.

To address whether FOCI formation was unique to strain PA99, we investigated type I pneumocyte injection using two additional strains. BL12 is a *P*. *aeruginosa* clinical isolate that was cultured from the blood of a patient with bacteremia, and PAK is a commonly used *P*. *aeruginosa* laboratory isolate. Unlike PA99, both BL12 and PAK naturally secrete ExoS and ExoT, and in this respect are typical of the *P*. *aeruginosa* isolates most commonly cultured from patients [11]. We introduced an *exoS* allele encoding enzymatically inactive ExoS (R146A and E379A/E381A substitutions resulting in loss of GAP and ADPRT activities, respectively) tagged with β-lactamase into a neutral site in the BL12 and PAK chromosomes (see Materials and Methods section). The resulting strains were designated BL12+S(R146A/E379A/E381A)bla and PAK+S(R146A/E379A/E381A)bla. In this way, we could identify injected cells using the TissueFAXS imaging system without affecting the normal progression of the infection [27,28]. The lungs were incubated with CCF2-AM, sectioned, stained for the type I pneumocyte marker, and analyzed using the TissueFAXS imaging system. BL12 caused a very severe pneumonia in mice, causing them to succumb prior to 23 hr, so results were only available for 12 or 18 hr. With this isolate, type I pneumocytes were injected earlier (at 12 hr) and to higher levels (>80% of injected cells at 23 hr) than with PA99Sbla (Fig 6A). As a result, FOCI were readily observable even at the 12 hr time point (Fig 6B). PAK likewise injected approximately 80% of type I pneumocytes by 23 hr (Fig 7A), and FOCI were observed at this time point (Fig 7B). These findings indicate that FOCI formation is not unique to strain PA99Sbla and suggest that *P*. *aeruginosa* strains differ in the rate and extent to which they form FOCI.

### ExoT is not sufficient to cause robust formation of FOCI during pneumonia

As mentioned, most clinical isolates of *P*. *aeruginosa* secrete both ExoS and ExoT. Since these effector proteins are quite similar (76% amino acid identity [29]), we examined whether ExoT was sufficient to cause the formation of FOCI. Technical difficulties prevented us from doing this in the PA99 background, so we used strain PAK. The *exoS* allele encoding enzymatically inactive ExoS tagged with β-lactamase was inserted into a neutral chromosomal site of strain PAKΔ*exoS*. This strain, designated PAKΔS+ S(R146A/E379A/E381A)bla, secreted ExoT but no enzymatically active ExoS (S9 Fig). It was compared to PAK+S(R146A/E379A/E381A)bla, which secreted both ExoS and ExoT. Mice were infected for 12 or 23 hr, at which times they were sacrificed and their lungs removed. Lungs were incubated with CCF2-AM, sectioned, stained for the type I pneumocyte marker, and analyzed using the TissueFAXS imaging system. In the absence of enzymatically active ExoS, PAK secreting ExoT injected relatively few type I pneumocytes (Fig 7A). Type I pneumocytes that were injected were relatively dispersed, and did not form readily apparent FOCI (Fig 7C). These findings indicate that ExoT by itself is not sufficient to cause robust formation of FOCI. This may be because ExoT has different enzymatic substrates than ExoS [30] or because bacteria secreting ExoS persist in higher numbers in the lungs than bacteria secreting only ExoT [18,31].

### ExoS-injected foci contain numerous dead cells

**Fig 8:**
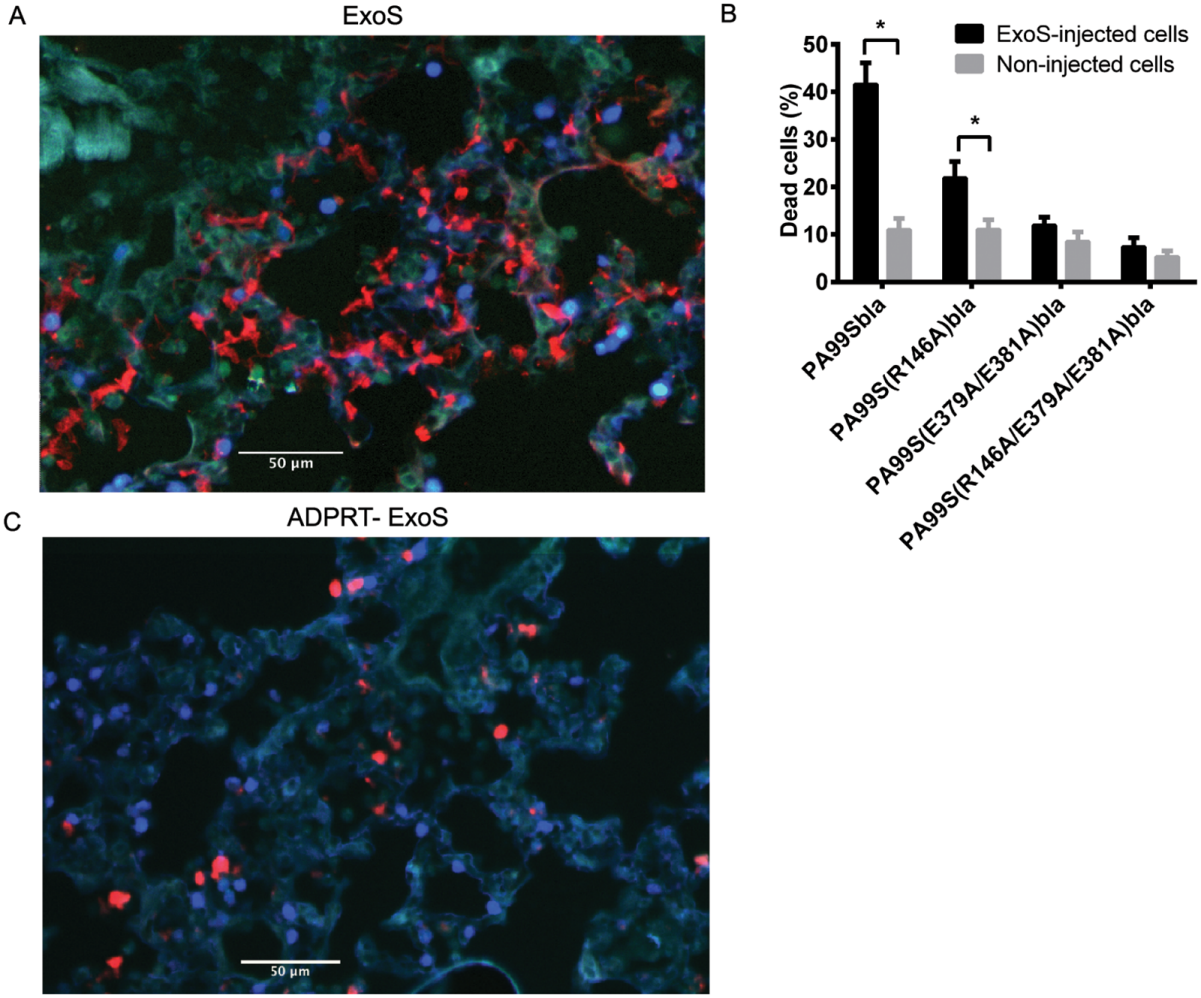
FOCI contain numerous dead cells. Mice were infected with PA99Sbla or strains secreting enzymatically inactive ExoS for 23 hr. The entire lung was incubated with a mixture of CCF2-AM and a dead cell stain and imaged with the TissueFAXS system. A) A focus of injected cells (blue) showing numerous dead cells (red) from a PA99Sbla-infected mouser. Scale bar represents 50 μm. B) For each lung lobe section, the proportions of dead cells among total ExoS-injected and total uninjected cells were counted. Data represent the average of at least 5 lobes pooled from 3 separate mice. Error bars represent standard deviations. (*, p < 0.05). C) A corresponding focus from the lungs of a mouse infected with PA99S(E379A/E381A)bla. Scale bar represents 50 μm.

**Fig 9:**
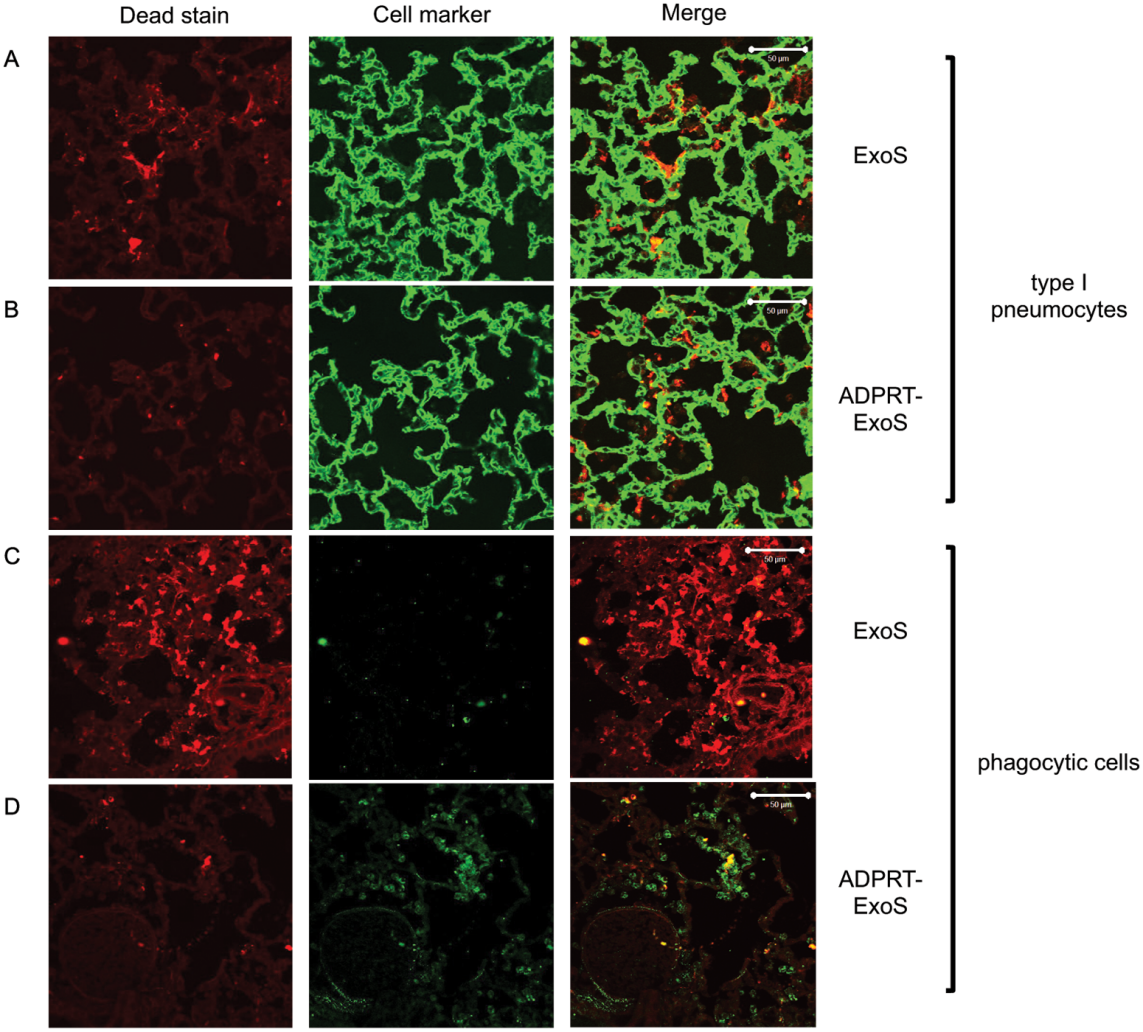
FOCI injected with ExoS contain many dead type I pneumocytes, whereas FOCI injected with ADPRT-deficient ExoS contain only a few dead phagocytic cells. Lungs infected with PA99Sbla (A & C) or PA99S(E379A/E381A)bla (B & D) were treated with a dead cell stain (red). Sections were additionally stained with either a caveolin-1 antibody to identify type I pneumocytes (A & B, green) or a Gr1 antibody to identify phagocytic cells (C & D, green). Dead cells from PA99Sbla-infected lungs were primarily type I pneumocytes. Relatively few dead cells were observed in sections from PA99S(E379A/E381A)bla-infected lungs, and these cells were usually phagocytes. Three sections from three different mice were examined for each cell marker; representative images are shown. Scale bars represent 50 μm.

ExoS is capable of causing cell death in cell culture systems [12,14,15,32]. We therefore characterized the viability of cells within FOCI. Using CCF2-AM in combination with a stain that identifies dead cells based upon membrane permeability, we found that many of the cells within FOCI were nonviable (Fig 8A). The majority of these cells had morphologies consistent with type I pneumocytes (Fig 8A). We confirmed the identity of these dead cells by staining lung sections with cell specific markers for type I pneumocytes (caveolin-1) and phagocytes (Gr1) in addition to the cell viability stain and performing confocal microscopy. The majority of non-viable cells within FOCI were indeed type I pneumocytes (Fig 9A), although some dead cells stained positive for a phagocytic cell marker (Fig 9C). These findings indicate that FOCI caused by ExoS injection contain large numbers of dead type I pneumocytes.

### The ADPRT activity of ExoS is necessary for rapid expansion of FOCI

We next investigated which of the enzymatic activities of ExoS were responsible for the formation of FOCI. We used strains of *P*. *aeruginosa* secreting ExoS with amino acid substitutions at residues critical for GAP activity (R146A) or ADPRT activity (E379A/E381A) or both GAP and ADPRT activities (R146A/E379A/E381A), and fused these variants with C-terminal β-lactamase tags. These strains were designated PA99S(R146A)bla, PA99S(E379A/E381A)bla, and PA99S(R146A/E379A/E381A)bla, respectively. Because the loss of ExoS enzymatic activity may result in more rapid bacterial clearance [18,22], we determined a dose for each mutant strain that yielded bacterial numbers in the lungs at 23 hr post-infection similar to those observed with wild type bacteria (S6 Fig). Mice were infected with the corresponding CFU of each mutant strain. At 23 hr post-infection, mice were sacrificed. The lungs were incubated with CCF2-AM, sectioned, stained for the type I pneumocyte marker, and analyzed using the TissueFAXS imaging system as described above. Lungs infected with the ADPRT-deficient strains PA99S(E379A/E381A)bla or PA99S(R146A/E379A/E381A)bla showed a trend towards smaller FOCI within the lungs (Fig 4B and 4C), suggesting that FOCI development and expansion was delayed during infection caused by these strains. As the infections progressed, enlarging FOCI associated with wild-type ExoS coalesced to encompass much of the lung lobe (Fig 4A), whereas those associated with ADPRT^-^ ExoS remained small and distinct (Fig 4C). These results indicate that the ADPRT activity of ExoS is necessary for the rapid expansion of FOCI during pneumonia.

Corresponding experiments were performed to examine the death of cells within FOCI. The use of a dead-cell stain demonstrated a higher proportion of death among injected cells in lungs infected with bacteria secreting fully active ExoS or GAP-deficient ExoS compared to bacteria secreting ADPRT-deficient or ADPRT/GAP-deficient variants of ExoS (Fig 8B). Visual inspection indicated that many of these dead cells were localized to FOCI (S5 Fig). Interestingly, although a substantial proportion of cells injected with GAP-deficient ExoS were killed, this proportion did not reach that observed with wild-type ExoS (Fig 8B), suggesting that ExoS GAP activity may also play a role in cell death. We next examined the identity of these dead cells. As stated previously, wild-type ExoS was primarily associated with the death of type I pneumocytes within FOCI, although some dead phagocytes were also noted (Figs 8A and 9). In contrast, relatively few dead cells were observed in FOCI associated with ADPRT-deficient ExoS-secreting bacteria, and those dead cells that were present had a rounded morphology, consistent with immune cells (Fig 8C). Cell type markers identified the majority of these cells as phagocytes (Fig 9D), while relatively few had markers of type I pneumocytes (Fig 9B). Together, these results indicate that the ADPRT domain of ExoS is responsible for the death of type I pneumocytes within FOCI during pneumonia.

It is conceivable that the ADPRT-negative variants of ExoS caused less type I pneumocyte cell death because they were preferentially injected into other cell types, not because they were less toxic. To examine this possibility, we measured the proportion of injected cells that were type I pneumocytes during pneumonia caused by bacteria secreting wild-type ExoS or the ExoS catalytic variants. At 23 hr post-infection, we did not observe a smaller proportion of type I pneumocytes injected with enzymatic variants of ExoS compared to wild-type ExoS (S7 Fig). In fact, the opposite was true. A higher proportion of type I pneumocytes was injected with enzymatically inactive ExoS, ADPRT-deficient ExoS, and GAP-deficient ExoS than was injected with fully active ExoS. This may have accounted for the overall increased blue fluorescence from sections of lungs injected with ADPRT-deficient ExoS compared to wild-type ExoS (Fig 8C) and is consistent with the enzymatic activities of ExoS rendering injected cells non-viable over time. The death and subsequent disintegration of cells injected with ADPRT^+^ ExoS would be expected to result in fewer intact injected cells in lung sections relative to cells injected with ADPRT^-^ ExoS.

### The ADPRT domain of ExoS contributes to heightened pulmonary-vascular leakage within the lungs

**Fig 10:**
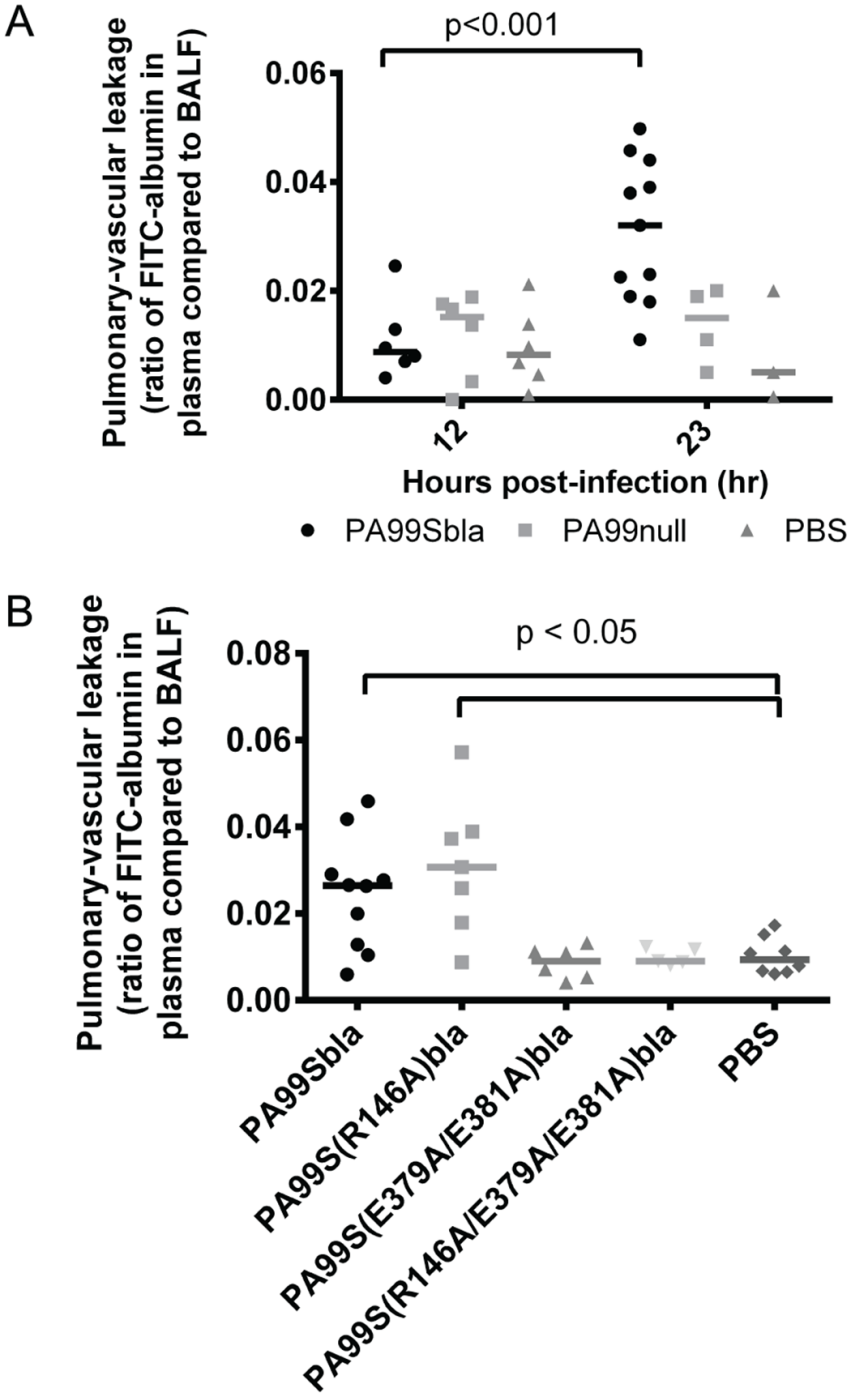
The ADPRT domain of ExoS contributes to disruption of the pulmonary-vascular barrier during pneumonia. Mice were inoculated with bacteria to cause pneumonia and then at 12 or 23 hours post-infection given FITC-albumin or saline control intranasally. Two hours later, cardiac punctures and bronchoalveolar lavages were performed to measure the amount of FITC fluorescence in the lungs and plasma. A) Mice were infected with PA99Sbla, PA99null, or PBS. B) Mice were infected with PA99Sbla (GAP^+^, ADPRT^+^), PA99S(R146A)bla (GAP^-^, ADPRT^+^), PA99S(E379A/E381)bla (GAP^+^, ADPRT^-^), or PA99S(R146A/E379A/E381A)bla (GAP^-^, ADPRT^-^). Each symbol represents a mouse. n ≥ 3 per condition. Bars indicate medians.

Bacterial dissemination from the lungs into the bloodstream is normally prevented by type I pneumocytes, which form the pulmonary-vascular barrier. Since ExoS is associated with bacterial dissemination during infection [17,18,33], we hypothesized that the formation of FOCI may be linked to disruption of this barrier and subsequent bacterial spread to the bloodstream. We therefore measured the integrity of the pulmonary-vascular barrier during pneumonia. Mice were infected with *P*. *aeruginosa* strains, and FITC-albumin was administered into their lungs by nasal aspiration. Leakage of the FITC-albumin into the bloodstream was then measured. As a control to determine what proportion of the disruption of the pulmonary-vascular barrier was due to ExoS and not other bacterial products or the overall inflammatory response, we used an isogenic non-secreting strain of *P*. *aeruginosa* designated PA99null. PA99null has a functional type III secretion system and is identical to PA99S except that it contains a deletion in the *exoS* gene. Since PA99null is rapidly cleared from the lungs [18,22,34], we adjusted the inoculum of this bacterium so that equivalent CFU of PA99null and PA99Sbla were present in the lungs at 23 hr post-infection (S6 Fig). At 12 hr post-infection, only background levels of FITC-albumin were detected in the blood of mice infected with either PA99Sbla or PA99null (Fig 10A). However, at 23 hr post-infection, greater amounts of FITC-albumin were detected in the blood of PA99Sbla-infected mice compared to PA99null-infected mice, indicating that ExoS contributed to disruption of the pulmonary-vascular barrier during infection.

We next investigated whether one or both of the enzymatic activities of ExoS was responsible for the pulmonary-vascular leakage. We repeated the FITC-albumin experiments using the strains secreting ExoS enzymatic variants. After controlling for bacterial CFU differences by adjusting the dose of each enzymatic mutant (S6 Fig), we found that an intact ADPRT domain of ExoS was associated for the heightened leakage of FITC-albumin into the blood (Fig 10B). These results indicate that the ADPRT activity of ExoS contributes to disruption of the pulmonary-vascular barrier.

### The ADPRT domain of ExoS contributes to bacterial dissemination to the blood during pneumonia

**Fig 11:**
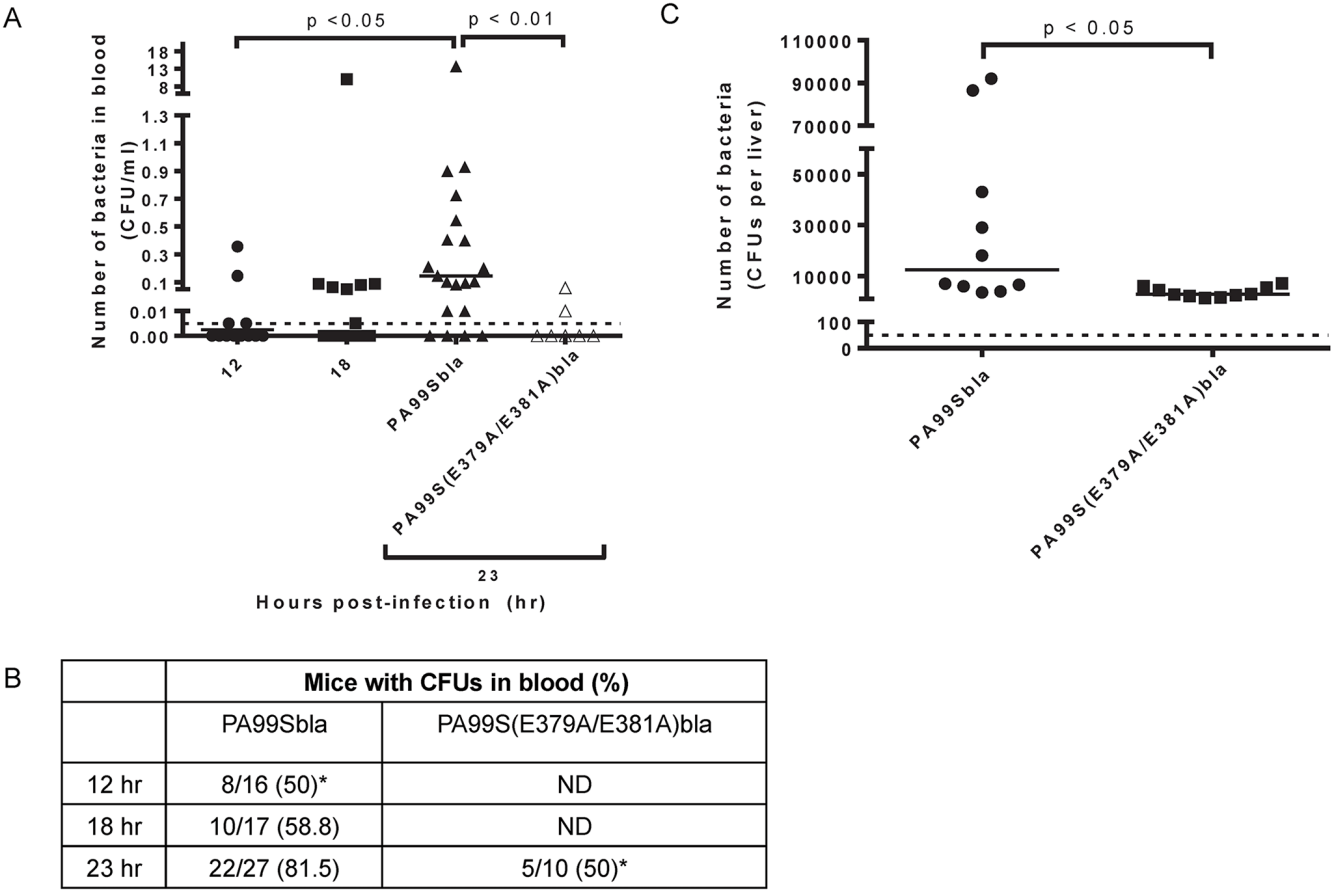
Bacterial dissemination into the bloodstream increases over time during pneumonia and is dependent on the ADPRT domain of ExoS. Mice were intranasally infected with PA99Sbla or PA99S(E379A/E381A)bla, and dissemination to the bloodstream was measured. The inoculum of PA99S(E379A/E381A)bla was adjusted such that equivalent CFU of this strain and PA99Sbla were recovered from the lungs at 23 hr post-infection. A) Number of bacteria in the blood. At 12, 18, or 23 hr post-infection, cardiac punctures were performed, and blood was plated for enumeration of bacteria. Each symbol represents one mouse. n ≥ 10 mice per strain. Bars indicate medians. Dotted line represents the limit of detection (0.005 CFU/μl). B) The proportion of mice with detectable bacteria in their blood. Samples were obtained as described in panel A. ND, not determined. (*, significantly different from dissemination frequency of PA99Sbla at 23 hr) C) Number of bacteria in the liver. At 23 hr post-infection, livers were removed, homogenized and plated. Each symbol represents one mouse. n = 10 per strain. Bars represent medians. Dotted line represents the limit of detection (50 CFU/liver).

Since our previous results indicated that ExoS disrupted the pulmonary-vascular barrier, we next wanted to examine whether this was linked to bacterial dissemination. We enumerated bacterial CFU in the blood of PA99Sbla-infected mice with pneumonia over time. As the pneumonia progressed, significantly higher densities of bacteria were detected in the blood of infected mice (Fig 11A). Likewise, bacteria were detected in a higher proportion of infected mice (Fig 11B). A functional ADPRT domain of ExoS was required for high levels of dissemination (Fig 11A and 11B). We also measured bacterial dissemination by quantifying bacterial numbers in the liver. Mice were infected with PA99Sbla or PA99S(E379A/E381A)bla, and bacterial counts in the liver were enumerated at 23 hr post-infection. Higher bacterial CFU in the liver were observed following infection with PA99Sbla compared to ADPRT-deficient PA99S(E379A/E381A)bla (Fig 11C). These results are in agreement with previous findings [18] and show that the ADPRT activity of ExoS contributes to bacterial dissemination to the blood and liver. However, bacteria were still detected in the blood of some mice infected with the ADPRT-deficient strain. It is plausible that other factors or activities allow dissemination to occur, albeit in a delayed manner.

## Discussion

Dissemination of bacteria from the lungs to the bloodstream bodes poorly for patients with pneumonia. Despite its clinical importance, the mechanisms by which bacteria escape the pulmonary compartment to spread systemically remain unclear. In the present study, we have explored this problem by focusing on *P*. *aeruginosa* and one of its major virulence determinants, ExoS. We used a novel imaging methodology to study the temporal and spatial aspects of ExoS injection into epithelial and immune cells in the context of a mouse model of pneumonia. We found that phagocytic cells, primarily neutrophils, were injected early during infection but that type I pneumocytes were injected at later time points. Injected type I pneumocytes were not observed throughout the lungs but were clustered into discrete regions (designated FOCI) that expanded as the pneumonia progressed. FOCI contained large numbers of dead cells, suggesting that they represent regions where the pulmonary-vascular barrier is compromised. Consistent with this hypothesis, pulmonary leakage of FITC-albumin and bacterial dissemination to the bloodstream both increased with expansion of FOCI. FOCI formation, pulmonary leakage, and bacterial dissemination all depended on the ADPRT activity of ExoS. We conclude that the ADPRT activity of ExoS facilitates spread of *P*. *aeruginosa* bacteria to the bloodstream following focal injection of ExoS into type I pneumocytes and subsequent disruption of the pulmonary-vascular barrier.

Injection of epithelial cells by ExoS had been previously demonstrated in vitro but not in vivo, primarily because these cells are difficult to collect by bronchoalveolar lavage or from excised lungs. In addition, the CCF2-AM injection reporter system requires the use of living cells and therefore cannot be readily applied to fixed tissue sections. For these reasons, current methodologies allow only limited assessment of the spatial distribution of injection in the lung. We used two innovations to overcome these difficulties. First, we employed the TissueFAXS imaging system, which allows analysis of an entire lung cross-section and, similar to cell sorters, quantifies the number of cells in the cross-section with specified staining characteristics. Second, we applied the CCF2-AM stain to intact lungs prior to fixation and sectioning. These modifications allowed us to discern spatial relationships between injected cells of various types, including type I pneumocytes. The combination of the TissueFAXS approach and the β-lactamase reporter assay allowed us to identify regions with large numbers of injected type I pneumocytes, which we have designated FOCI. This method may have utility in the study of other pulmonary pathogens as well as to other organ systems.

The factors dictating which regions of the lungs evolve into FOCI remain unclear. In the mouse model of pneumonia, bacteria spread rapidly throughout the lungs and are already found in the alveoli and distal airways at 3 hr post-infection [35]. However, bacteria are likely not uniformly distributed and may be more abundant in some regions of the lungs than others. Although our analysis did not quantify bacterial numbers, we did qualitatively observe higher concentrations of bacteria inside of FOCI than outside of FOCI (S5 Fig). Therefore FOCI may form in regions of the lung that receive an unusually large inoculum of bacteria. Alternatively, the increased bacterial numbers may be the result and not the consequence of FOCI. FOCI may foster higher bacterial numbers because their dead cells release nutrients that fuel increased bacterial replication or because the tissue damage in these regions impairs an effective immune response.

Interestingly, FOCI often contained small clusters in which injected type I pneumocytes were directly adjacent to one another (Fig 5F and S4 Fig). It may be that injection of a single type I pneumocyte initiates a process whereby neighboring type I pneumocytes become prone to subsequent injection as the result of a breach in the polarized nature of the monolayer. Such a positive-feedback process would then result in rapidly enlarging clusters of injected cells. Interestingly, discreet plaque-like areas of injected cells have been described in several cell culture systems using primary human, mouse airway epithelial cells, or canine kidney epithelial cells [36–39]. Our findings suggest that FOCI formation may be an in vivo correlate of this in vitro phenomenon.

Although ExoS has both GAP and ADPRT activities, we found that the ADPRT activity was primarily responsible for robust FOCI formation and bacterial dissemination. Others have similarly noted that the GAP activity of ExoS plays at best a minor role in the disruption of endothelial and epithelial cell monolayers in vitro [39,40]. Following infection with a mutant secreting an ADPRT-deficient form of ExoS, type I pneumocytes were still injected, but the resulting FOCI remained small. In addition, relatively few dead type I pneumocytes were present in these FOCI. Thus rapid expansion of FOCI requires the ADPRT activity of ExoS, although other mechanisms may suffice for the formation of FOCI. It remains unclear which of the phenotypes associated with the ExoS ADPRT activity (e.g. cell rounding, apoptosis, bleb-niche formation, or anti-internalization [12–16,41]) is responsible for the formation of FOCI.

Whereas large numbers of neutrophils were already injected with ExoS by 6 hr post-infection, relatively few type I pneumocytes were injected with this effector protein even at 12 hr post-infection. Several explanations may account for the delayed injection of type I pneumocytes relative to neutrophils. Neutrophils and other phagocytic cells may be preferentially targeted for rapid injection by the *P*. *aeruginosa* type III secretion system, as is the case for the closely related *Yersinia pseudotuberculosis* type III secretion system [42,43]. The active chemotaxis of phagocytic cells towards bacteria may allow them to more rapidly achieve direct contact with bacteria, a prerequisite for type III secretion. Alternatively, bacteria may be prevented from contacting type I pneumocytes early during infection by the protective layer of surfactant that coats the alveoli and which may form a physical barrier that the type III secretion needle cannot penetrate. As the infection progresses, this protective layer may be compromised by *P*. *aeruginosa* products such as elastase B or host inflammatory factors such as cathepsin G, neutrophil elastase, and proteinase-3, all of which degrade surfactant protein SP-A [44,45]. Perhaps the most likely explanation is that the polarized nature of type I pneumocytes establishes an apical barrier that is relatively resistant to *P*. *aeruginosa* attachment and type III injection [46,47].

We focused on the role of ExoS in facilitating bacterial dissemination to the bloodstream, but disruption of the pulmonary-vascular barrier may also adversely affect disease outcomes in other ways. In vitro studies indicate that ExoS causes loss of focal adhesions and retraction of endothelial cells [40,48]. Although we did not examine endothelial cells in our study, disruption of the epithelial barrier has been associated with corresponding disruption of the endothelial barrier in pneumonia caused by ExoS^+^ *P*. *aeruginosa* [17]. Such effects on endothelial cells could further contribute to the disruption of the overall pulmonary-vascular barrier and lead to prolonged alveolar edema [49–51], which may potentiate severe pneumonia [49,52]. Of note, *P*. *aeruginosa* pneumonia in humans is accompanied by a high incidence of acute lung injury, the clinical correlate of destruction of the alveolar epithelia and enhanced permeability of the pulmonary-vascular barrier [49–51]. Thus ExoS injection into pulmonary epithelial cells may play an important role in the progression of pneumonia.

We propose the following model to explain the role ExoS in bacterial dissemination during pneumonia. Bacteria enter the lungs and cause a rapid recruitment of inflammatory cells, primarily neutrophils. Early during infection, neutrophils are the predominant cell type injected with ExoS, which inhibits phagocytosis and prevents bacterial clearance [12,22]. As the infection progresses, small, discrete foci of type I pneumocytes become injected. The ADPRT activity of ExoS targets host cell substrates essential for maintenance of type I pneumocyte tight junctions and viability. This in turn leads to expansion of the foci and compromise of the pulmonary-vascular barrier. As a result, bacteria disseminate from the lungs into the bloodstream. The net effects are the worse clinical outcomes associated with ExoS^+^ *P*. *aeruginosa* infections. This work highlights one example of how a pulmonary pathogen utilizes a effector protein to promote dissemination to the bloodstream. Additional studies are necessary to determine whether other respiratory pathogens use similar mechanisms to escape from the lungs during pneumonia.

## Materials and Methods

### Bacterial strains and growth conditions

The *P*. *aeruginosa* clinical isolate PA99 secretes ExoU, ExoS, and ExoT [11,53]. This strain was chosen for the present study because it is well characterized in the context of the mouse model of pneumonia and because it allows for the comparison of the effects of ExoS to those of ExoU in an isogenic background [18,22,31,34,54–56]. PA99S was previously derived from PA99 by disruption of the *exoU* and *exoT* genes [11]. PA99null, which possess an intact type III secretion apparatus but does not secrete known effector proteins, was previously generated by disrupting all three effector genes (*exoU*, *exoS*, and *exoT*) [18]. Also previously generated was PA99*ΔpscJ*, a strain identical to PA99 except that it has a disrupted *pscJ* gene, which renders its type III secretion apparatus defective [18]. A strain complemented for ExoS secretion, designated PA99null+S, was previously generated by integrating the *exoS* gene with its promoter into a neutral site in the chromosome using plasmid mini-CTX*exoS* [18]. PAK is a laboratory strain of *P*. *aeruginosa* that secretes ExoS and ExoT [57]; PAKΔS secretes only ExoT due to disruption of the *exoS* gene [15]. BL12 is a clinical isolate cultured from the blood of a patient at Northwestern Memorial Hospital, Chicago, IL. It secretes ExoS and ExoT (Fig 9A). Further details on strains and plasmids used in this study can be found in S1 Table.

Bacterial strains were streaked from frozen cultures onto Vogel-Bonner minimal (VBM) agar [58]. Overnight cultures were grown in 5 ml MINS medium [59] at 37°C. Cultures were diluted into fresh medium the next day and re-grown to exponential phase prior to infections.

### Generation of strains secreting ExoS-β-lactamase fusion proteins

The stop codon of the *exoS* gene in plasmid mini-CTX*exoS* [18] was removed by site-directed mutagenesis with primers (5’- GGCCTTGATCTGGCCGGACCGGTCGTAAA-3’) and (5’- CGTCTTTCTTTTACGACCGGTCCGGCCAGAT-3’) to generate mini-CTX*exoS*Δstop. The DNA fragment encoding TEM-1 β-lactamase was amplified from pBR322 by PCR using primers with engineered AgeI sites as previously described [54]. This fragment was ligated into mini-CTX*exoS*Δstop to generate mini-CTX*exoSbla*, which encodes for a translational fusion of ExoS and the TEM-1 β-lactamase (“ExoS-Bla”). This plasmid was then transformed into *E*. *coli* S17.1 and conjugated into the PA99null chromosome via integrase-mediated recombination at the *attB* site as previously described [60] to generate PA99Sbla. mini-CTX*exoSbla* was also introduced into the chromosome of PA99ΔpscJ [18] to create PA99ΔpscJ+Sbla, a strain identical to PA99Sbla except that it has a disrupted type III secretion apparatus. Proper secretion, translocation, and/or cytotoxic activity of tagged ExoS-Bla were confirmed by immunoblot analyses, cell death assays, CCF2-AM translocation assays, and/or bacterial persistence studies using the mouse model of pneumonia (S8 and S9 Figs).

To generate enzymatically inactive ExoS variants with β-lactamase tags, site-directed mutagenesis was performed on mini-CTX*exoS-bla* using primer sets as previously described [18]. In this way, we generated plasmids mini-CTX*exoS(R146A)bla*, mini-CTX*exoS*(*E379A/E381A)bla*, and mini-CTX*exoS(R146A/E379A/E381A)bla* [27,28]. These plasmids were then transformed into *E*. *coli* S17.1 as described above to generate PA99S(R146A)bla, which is GAP^-^, PA99S(E379A/E381A)bla, which is ADPRT^-^, and PA99S(R146A/E379A/E381A)bla, which is both GAP^-^ and ADPRT^-^. mini-CTX*exoS(R146A/E379A/E381A)bla* was complemented into PAK, PAKΔS, and BL12 to study ExoS injection in the context of these strain backgrounds. In this way, we generated PAK+S(R146A/E379A/E381A)bla, PAKΔS+ S(R146A/E379A/E381A)bla, and BL12+S(R146A/E379A/E381A)bla. Proper secretion and translocation of the ExoS-bla fusion protein by these strains was confirmed using immunoblot analysis and a fluorescence cell assay (S9 Fig).

### Mouse model of acute pneumonia

The mouse model of acute pneumonia described by Comolli *et al*. [61] was used for all animal experiments. Briefly, 6- to 8- week-old female BALB/c mice were anesthetized by intraperitoneal injection of a mixture of ketamine (75 mg/kg) and xylazine (5 mg/kg). Mice were intranasally inoculated with 4.6–9.2 x 10^6^ CFU PA99Sbla or PA99S(R146A)bla, or 1.8 x 10^7^ CFU PA99S(E379A/E381A)bla, PA99S(R146A/E379A/E381A)bla, or PA99null in phosphate buffered saline (PBS). These doses led to equivalent CFU of bacteria in the lungs at 23 hr post-infection (S6 Fig). Inocula were confirmed by plating serial dilutions on VBM agar for enumeration. At appropriate times post-infection, the mice were anesthetized and sacrificed by cervical dislocation. For dissemination experiments, organs were excised and homogenized in PBS. Viable bacteria were enumerated by plating serial dilutions on VBM agar. For quantifying bacterial CFUs within blood, cardiac punctures were performed immediately post-mortem and the obtained blood placed into tubes containing 5 USP units of heparin. Bacteria were enumerated by plating 100 μl aliquots on VBM agar.

Animals were purchased from Harlan Laboratories, Inc. (Indianapolis, IN) and housed in the containment ward of the Center of Comparative Medicine at Northwestern University. All experiments were approved by the Northwestern University Animal Care and Use Committee.

### Analysis of ExoS injection by flow cytometry

To remove circulating blood, the vasculatures of lungs were flushed post-mortem by injection of 2 ml PBS into the right ventricle of the heart. Lungs were excised, pressed through 40-μm filters (BD Falcon, Becton, Dickinson and Company, Franklin Lakes, NJ), and rinsed repeatedly with PBS. The recovered cells were pelleted by centrifugation at 500 x *g* for 5 min at 4°C. Red blood cells were lysed by adding 5 ml of cold, sterile H_2_O and gently shaking for 30 sec. Five milliliters of 2X normal saline (1.8% NaCl) was added to stop lysis. The remaining cells were pelleted and resuspended in 1 ml PBS. Viable cells were quantified using a hemocytometer by counting cells that excluded trypan blue. A total of 2 x 10^5^ cells were pelleted and resuspended in 100 μl PBS containing 1X CCF2-AM loading solution (Invitrogen). Cells were incubated in the dark for 1 hr at room temperature. Cells were then pelleted and incubated with 10% rat serum (1:4 final dilution) (Sigma-Aldrich, St. Louis, MO) and anti-CD16/32 (1:4 final dilution) in fluorescence-activated cell sorting (FACS) buffer (1% BSA, 1 mM EDTA in PBS) for 15 min on ice. To identify individual cell types, cells were incubated in the dark with cell-discriminatory antibodies at appropriate dilutions (see below) in 125 μl FACS buffer for 30 min. Cells were pelleted, fixed in 3.7% paraformaldehyde in PBS for 2 min, and washed twice with FACS buffer. Final cell suspensions were filtered through 80-μm nylon mesh (Small Parts Inc., Miami Lakes, FL) into 12 x 75 mm round-bottom tubes (BD Falcon). Final antibody dilutions were as follows: anti-CD16/32, 1:50; anti-CD45, 1:1,500, anti-Gr1, 1:1,500; anti-F4/80, 1:50; anti-CD4, 1:500; anti-CD8, 1:500; anti-CD19, 1:500; anti-CD49, 1:500; anti-CD11b, 1:2,500; anti-CD11c, 1:500; and isotype controls, 1:100 each. All antibodies were purchased from eBioscience (San Diego, CA).

Analysis was performed on the BD FACSCanto II flow cytometer (Becton, Dickinson and Company) and analyzed using FlowJo version 8.8.6 software (Tree Star, Inc., Ashland, OR). Immune cells were gated as follows: CD11b^+^Gr1^hi^, neutrophils; CD11b^+^Gr1^int^, recruited monocytes; Gr1^-^F4/80^+^, resident macrophages; CD11c^int^Gr1^-^, dendritic cells; CD4^+^, helper T cells; CD8^+^, cytotoxic T cells; CD19^+^, B cells; and CD49^+^, NK cells. The total number of viable inflammatory cells per mouse lung was determined by equating the number of viable inflammatory cells measured on the flow cytometer (using forward and side scatter properties) to the number of trypan blue-negative cells counted on the hemocytometer.

### Staining of tissue sections for ExoS injection and cell viability

Lungs were excised and instilled with 800 μl of 6X CCF2-AM loading solution (Life Technologies, Carlsbad, CA) and then placed in a vial of 3 ml 6X CCF2-AM loading solution for 1 hr at room temperature in the dark. For cell viability experiments, LIVE/DEAD Fixable Red Dead Cell stain (Life Technologies) was added to the CCF2-AM mixture at a final dilution of 1:500. The lungs were then moved to 10 ml of 4% paraformaldehyde and incubated overnight at room temperature. Lungs were cryoprotected by incubation in 15% sucrose solution for 8 hr and 30% sucrose solution overnight. Lungs were frozen using Clear Frozen Section Compound (VWR, Radnor, PA) in a dry ice/isopentane bath. Sections (6 μm thickness) were cut by the Mouse Histology and Phenotyping Core of the Robert H. Lurie Comprehensive Cancer Center at Northwestern University. Slides were stored at -80°C prior to additional staining.

For visualization of cell types, slides were acclimated to room temperature and blocked by incubation in 10% mouse serum, 1% BSA in Tris-buffered saline (TBS) for 2 hr. Slides were then incubated overnight at 37°C in a humidified chamber with 1:1,000 dilutions of primary antibodies in 1% BSA in TBS. Primary antibodies to caveolin-1 (Abcam, Cambridge, England), pSP-C (Abcam), or Gr1 (Abcam) were used to detect type I pneumocytes, type II pneumocytes, and phagocytes (neutrophils and monocytes), respectively. (Note that Gr1^-^ resident macrophages comprise a very small fraction of phagocytic cells in the lungs at 12 hr and later during infection [22].) For detection of type I and type II pneumocytes, slides were washed twice with 0.05% Triton X-100 in TBS for 5 min and then incubated with AlexaFluor 555-conjugated goat anti-rabbit secondary antibody (Molecular Probes, Eugene, OR) diluted 1:1,000 in 1% BSA in TBS for 1 hr at 37°C. No secondary antibody was necessary for the detection of phagocytes, as the primary Gr1 antibody was conjugated to Cy5. For visualization of bacteria, slides were blocked as described above and then incubated with 1:1,000 diluted primary antibodies to clinical isolate PA99 [56] for 2 hr at 37°C. Slides were washed twice with 0.05% Triton X-100 in TBS for 5 min and then incubated with 1:1,000 diluted AlexaFluor 555-conjugated goat anti-rabbit secondary antibody for 1 hr at 37°C. Slides were washed twice for 5 min with 0.05% Triton X-100 in TBS, air-dried, and mounted using ProLong Gold antifade mounting media (Molecular Probes). Images were acquired at 200X using the TissueFAXS imaging system (TissueGnostics, Vienna, Austria) located in the Cell Imaging Facility at Northwestern University.

### Analysis of lung sections

Analysis was performed using the TissueQuest 4.1 software (TissueGnostics USA Ltd., Tarzana, CA). This technique employs a cellular reconstruction algorithm called microscopy-based multicolor tissue cytometry [62,63]. In this algorithm, high and low fluorescent intensity thresholds are used to filter each pixel. If a pixel falls within these thresholds, it is considered valid, and it is grouped with all directly adjacent valid pixels to form an object. The object is enlarged by continued assessment and addition of adjacent pixels until no further adjacent pixels are found that meet the criteria for validity. At that point, the resulting object is designated a cell [62]. Pixels are evaluated across an entire lung cross-section. In this way, all cells in a lung cross-section are counted. At least four mice were analyzed per time point, with two lobes chosen at random from each mouse. Due to limitations of the fluorescence spectral overlap of CCF2-AM, only one primary antibody could be imaged along with CCF2-AM per slide. Three adjacent 6 μm lung sections per mouse lung were therefore used to analyze for injected type I pneumocytes, type II pneumocytes, and phagocytic cells. Lung sections infected with PA99null+S (no β-lactamase tag) were used to determine baseline blue:green fluorescence ratio thresholds for each cell type (≥ 1.3 for type I pneumocytes and phagocytic cells; ≥1.1 for type II pneumocytes), since all cells in these sections lack blue fluorescence from cleaved CCF2-AM. Injected cells from lung sections infected with bacteria secreting β-lactamase-tagged ExoS were identified as those cells with a blue:green fluorescence ratio exceeding these baseline thresholds. In this way, the total population of ExoS-injected cells in a tissue cross-section of an entire lung lobe was determined. These cells were then subdivided into populations of ExoS-injected cells staining positive for each individual cell type marker. A schematic overview of this process is provided in S2 Fig. TissueQuest software was used to analyze the spatial distribution of ExoS-injected cells within lung sections. The average area of FOCI in a lung section was calculated by manually drawing a border around each region of the tissue section that contained a high density of injected cells, calculating the area of each FOCI in the lung section, summing all these areas, and dividing the total area by the total number of FOCI in the lung section.

To quantify the number of dead cells, the percentages of injected and uninjected cells that stained positive with the LIVE/DEAD Fixable Red Dead Cell stain were determined. TissueQuest software was used to count the total number of ExoS-injected and non-injected cells per lung lobe. Then the number of those cells also positive for the DEAD stain was determined to calculate the final percentages of dead cells. To determine which cell types were dead, slides were stained with antibodies that recognized cell discriminatory markers as described above. Imaging was performed on a Zeiss UV LSM510 confocal microscope in the Nikon Cell Imaging Facility at Northwestern University.

### Quantifying pulmonary-vascular leakage

Mice were anesthetized and intranasally administered 50 μl FITC-conjugated albumin (Sigma) at a concentration of 50 mg/ml in 0.9% saline. Two hours after the FITC-albumin administration, mice were anesthetized and sacrificed by cervical dislocation. Blood was collected by cardiac puncture, and BALF was collected by twice instilling and withdrawing 1 ml PBS. The collected blood was centrifuged at 4000 x *g* for 10 min to separate plasma from red blood cells. FITC fluorescence of the BALF and plasma were measured using a SpectraMax M3 fluorescence plate reader (Molecular Devices, Sunnyvale, CA). A standard curve of FITC-albumin fluorescence vs. concentration was generated and used to determine the FITC-albumin concentration in each sample. The ratio of FITC-albumin concentration in the blood to that in the BALF was calculated.

### Statistical analysis

Statistical analysis was performed using GraphPad Prism 6 (GraphPad Software, Inc., La Jolla, CA). An analysis of variance (ANOVA) followed by Bonferroni correction for multiple comparisons was performed for injection, foci measurement, cell viability, and pulmonary-vascular leakage experiments. The Kruskal-Wallis comparison of medians followed by Dunn’s test for multiple comparisons was performed for blood CFU experiments. Fisher’s exact test was performed on frequencies of dissemination. A p-value of 0.05 was used as a threshold for significance.

## Supporting Information

S1 Fig The TissueFAXS/TissueQuest system identifies ExoS-injected cells and discriminates type I pneumocytes, type II pneumocytes, and phagocytes in lung sections. Following 18 hr of infection with PA99Sbla, the lungs were removed, stained with CCF2-AM, fixed, and frozen prior to sectioning. A) An entire lung lobe stained with CCF2-AM. Blue fluorescent cells represent injected cells; green fluorescent cells represent uninjected cells. Scale bar equals 1 mm. The contrast and brightness for all color filters were uniformly adjusted over the entire image for better visualization. B-E) Higher magnification views of the same lobe stained with B) CCF2-AM, C) caveolin-1/Alexa Fluor 555 for identification of type I pneumocytes, D) pSP-C/Alexa Fluor 555 for identification of type II pneumocytes, and E) Gr1/Cy5 for identification of phagocytic cells. For panels B-E, scale bars represent 50 μm.
(TIF)

S2 Fig The TissueFAXS imaging system and TissueQuest software allow calculation of the proportion of each cell type injected with ExoS. To determine levels of background fluorescence, mice were infected with a *P*. *aeruginosa* strain secreting untagged ExoS (no β-lactamase). Adjacent lung tissue sections were stained with CCF2-AM and one of the cell type markers (caveolin-1/Alexa Fluor 555 for type I pneumocytes, pSP-C/Alexa Fluor 555 for type II pneumocytes, or Gr1/Cy5 for phagocytic cells). Tissue sections were imaged using the TissueFAXS imaging system. TissueQuest software was then used to measure the fluorescence of each cell in the tissue sections. For each cell type (type I pneumocytes, type II pneumocytes, phagocytic cells), blue:green fluorescence ratio thresholds were determined that excluded the majority of cells exhibiting background fluorescence. Next, mice were infected with a *P*. *aeruginosa* strain that secreted β-lactamase tagged ExoS. Lung tissue sections were similarly processed. The blue:green fluorescence ratio of each cell in the tissue section was measured, and cells with a fluorescence ratio that exceeded the previously defined thresholds were scored as “injected” and counted. Each adjacent tissue section was analyzed for injected cells and one of the cell type markers to determine the proportion of injected cells in that tissue section that were of that particular cell type (e.g. the proportion of injected cells that were type I pneumocytes).
(EPS)

S3 Fig The distribution of ExoS-injected cells within lung sections was determined using the TissueFAXS imaging system and TissueQuest software. ExoS-injected cells were distinguished from uninjected cells in lung sections by gating for the appropriate blue:green fluorescence ratios for each cell type and then marking those injected cells on the original image. A) A blue-gray scale image of a representative lung section at 18 hr post-infection. Injected cells of any type were identified by their high blue fluorescence intensities (white cells). Cell type specific markers were subsequently used to identify the type of each injected cell. In this example, those injected cells that were identified as type I pneumocytes by caveolin-1 antibody staining are outlined in red. Scale bar represents 20 μm. B) A lobe from the lung of a mouse infected with PA99Sbla following staining with CCF2-AM. Scale bar represents 500 μm. One region of the lung demonstrating substantial amounts of blue fluorescence is outlined in white. C) Higher magnification view of the outlined region in panel B. A high density of blue fluorescent cells, which represent those cells injected with ExoS, is observed. D) The same image as shown in panel C but with ExoS-injected cells identified by the TissueQuest software and marked with white outlines. Scale bars in panels C and D represent 100 μm.
(EPS)

S4 Fig FOCI consist of clusters of type I pneumocytes. Each panel represents a FOCI and is taken from the white boxes shown in Fig 5. Type I pneumocytes (caveolin-1^+^ cells) are outlined in white. A) 12 hr post-infection. B) 18 hr post-infection. C) 23 hr post-infection. Scale bars represent 100 μm.
(TIF)

S5 Fig Bacteria are located both within and outside of FOCI. Tissue sections of lungs obtained at (A) 12 hr and (B & C) 23 hr post-infection with PA99Sbla were stained for bacteria (red) using the TissueFAXS imaging system. Shown are representative images inside (A & B) and outside (C) FOCI at each time point. Scale bars represent 100 μm.
(EPS)

S6 Fig Adjustment of inocula of different bacterial strains to achieve similar CFU in the lungs of mice at 23 hr post-infection. Mice were infected with 4.6 x 10^6^–9.2 x 10^6^ CFU PA99Sbla or PA99S(R146A)bla, 1.8 x 10^7^ CFU PA99S(E379A/E381)bla, 1.8 x 10^7^ CFU PA99S(R146A/E379A/E381A)bla, or 1.8 x 10^7^ PA99null bacteria. At 23 hr post-infection, lungs were removed, homogenized and plated. The average CFU of ExoS mutant strains recovered from whole lungs of mice were normalized to the number of CFU of PA99Sbla recovered at the same time point. Error bars represent SEM. n ≥ 3 per strain.
(TIF)

S7 Fig Type I pneumocytes are injected with ExoS variants lacking GAP and/or ADPRT activity. The proportion of injected cells that were type I pneumocytes varied with the enzymatically inactive form of ExoS secreted by the infecting bacteria. Lungs were harvested at 23 hr post-infection. Each symbol represents the value measured from a cross-section of an entire lung lobe. At least 6 lobes were analyzed per strain. Bars indicate medians.
(TIF)

S8 Fig The β-lactamase tag does not detectably alter ExoS secretion, translocation, or activity. A) Secretion of tagged and untagged ExoS. Proteins in supernatants from overnight cultures of PA99S, PA99Sbla, and PA99*ΔpscJ*-Sbla were precipitated and used in immunoblot analyses with antibodies that recognized ExoS. PA99*ΔpscJ*-Sbla produces an ExoS-Bla fusion protein but lacks a functional type III secretion apparatus to secrete it. It was used as a control to ensure ExoS-Bla protein in the culture supernatant was secreted and not released from dying bacteria. Since type III secreted effector proteins comprise a large proportion of the proteins exported by *P*. *aeruginosa*, each supernatant sample was normalized to the same volume. B) Numbers in mouse lungs of bacteria secreting tagged and untagged ExoS. A strain secreting ExoS-Bla persists in the lungs at approximately the same levels as a strain secreting untagged ExoS. Mice were infected with PA99S or PA99Sbla. After 24 hr, lungs were removed and bacterial CFUs were enumerated by plating. Means are plotted. n = 3 mice per strain. Error bars represent SEM. C) Translocation of tagged and untagged ExoS. Since ExoS must be injected into mammalian cells to cause cell death, we used cell lysis to quantify ExoS translocation. HeLa cells were infected with an MOI of 10 with PA99S, PA99Sbla, or PA99S(E379A/E381A) (ADPRT^-^) for 8 hr. Lactate dehydrogenase (LDH) levels in the medium were quantified to determine cell lysis. Levels of cell lysis were normalized to a Triton-treated control. *, significantly lower than PA99S. PA99S and PA99Sbla were not significantly different.
(EPS)

S9 Fig ExoS(R146A/E379A/E381A)bla is appropriately secreted and translocated from strains PAK and BL12. A) Proteins in supernatants from overnight cultures of PAK, PAK+S(R146A/E379A/E381A)bla, PAKΔS, PAKΔS+ S(R146A/E379A/E381A)bla, BL12, and BL12+S(R146A/E379A/E381A)bla were precipitated and used in immunoblot analyses with antibodies that recognized ExoS and ExoT. Strains complemented with the ExoS-Bla fusion protein show proper secretion of the larger protein. B) J774 macrophage-like cells were infected for 3 hr with various strains of *P*. *aeruginosa* secreting ExoS-Bla or ExoS(R146A/E379A/E381A)bla. Cells were incubated with CCF2, and translocation was quantified using a fluorescence plate reader. A high ratio of blue:green fluorescence indicates translocation of the β-lactamase tagged ExoS (*, p < 0.05 in comparison to uninfected cells).
(TIF)

S1 Table Bacterial strains and plasmids. (DOCX)

S1 File Supplemental materials and methods. (DOCX)
