# Correction: The Role of ExoS in Dissemination of *Pseudomonas aeruginosa* during Pneumonia

**DOI:** 10.1371/journal.ppat.1005163

**Published:** 2015-09-17

**Authors:** Stephanie M. Rangel, Maureen H. Diaz, Claire A. Knoten, Angelica Zhang, Alan R. Hauser

In the "merge" panel of Fig 9, row B, the authors inadvertently inserted the incorrect image. The corrected version of this figure is provided here.

**Fig 9:**
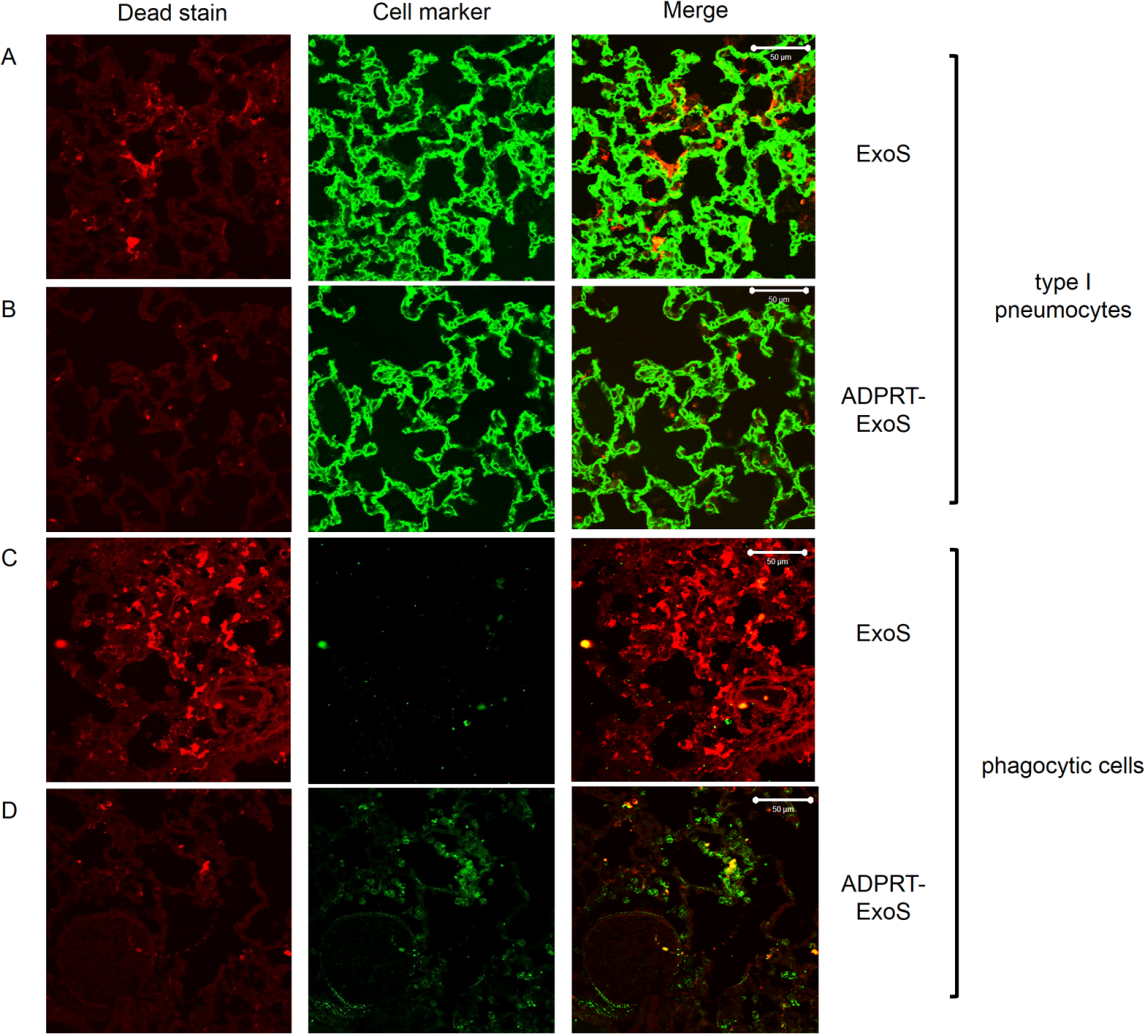
FOCI injected with ExoS contain many dead type I pneumocytes, whereas FOCI injected with ADPRT-deficient ExoS contain only a few dead phagocytic cells. Lungs infected with PA99Sbla (A & C) or PA99S(E379A/E381A)bla (B & D) were treated with a dead cell stain (red). Sections were additionally stained with either a caveolin-1 antibody to identify type I pneumocytes (A & B, green) or a Gr1 antibody to identify phagocytic cells (C & D, green). Dead cells from PA99Sbla-infected lungs were primarily type I pneumocytes. Relatively few dead cells were observed in sections from PA99S(E379A/E381A)bla-infected lungs, and these cells were usually phagocytes. Three sections from three different mice were examined for each cell marker; representative images are shown. Scale bars represent 50 μm.
