# Supplementary figures and images for: The Role of ExoS in Dissemination of Pseudomonas aeruginosa during Pneumonia
Source: PLoS Pathog. 2015 Jun 19;11(6):e1004945. doi: 10.1371/journal.ppat.1004945 (PMC4474835; doi:10.1371/journal.ppat.1004945)

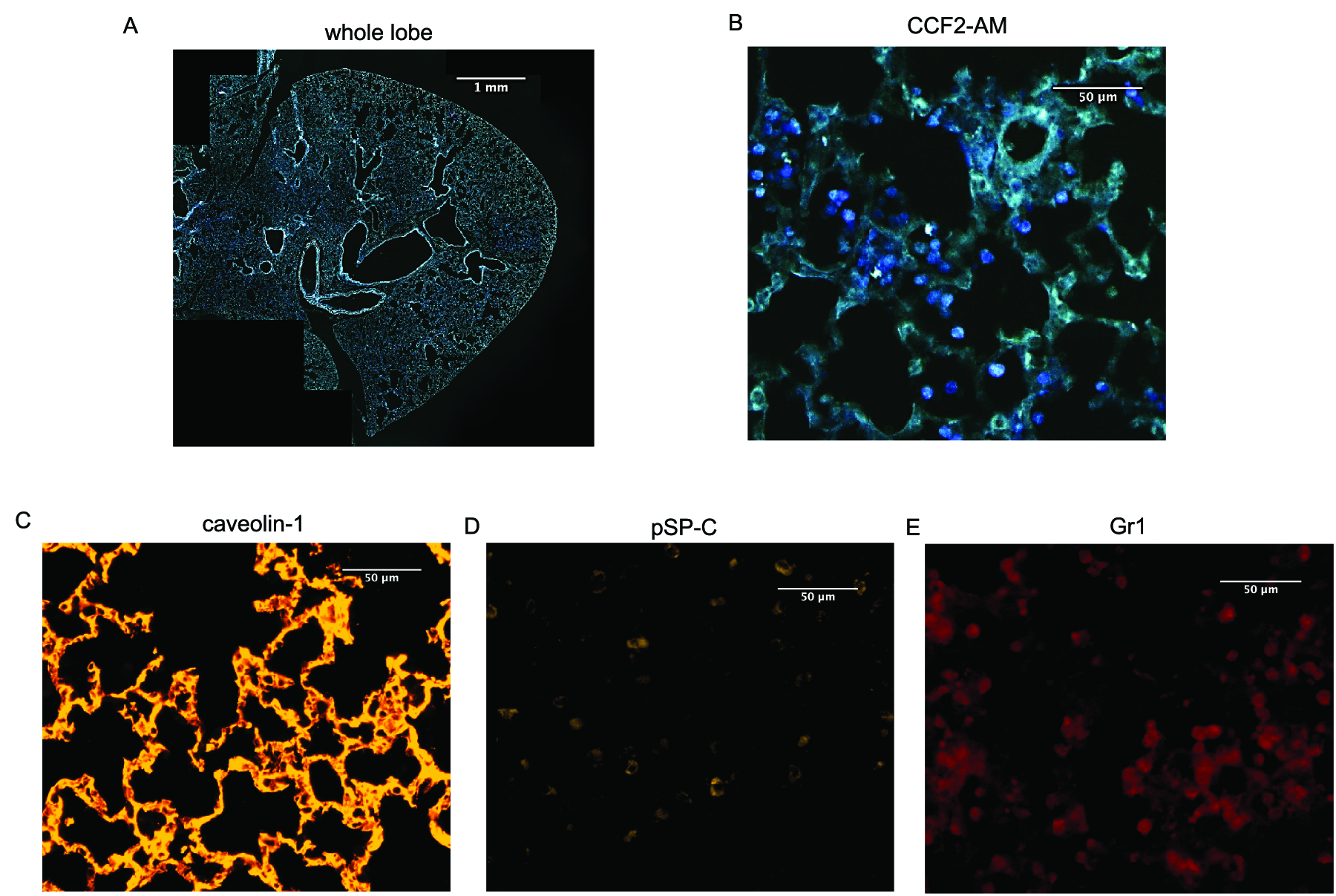

Supplement: S1 Fig — Following 18 hr of infection with PA99Sbla, the lungs were removed, stained with CCF2-AM, fixed, and frozen prior to sectioning. A) An entire lung lobe stained with CCF2-AM. Blue fluorescent cells represent injected cells; green fluorescent cells represent uninjected cells. Scale bar equals 1 mm. The contrast and brightness for all color filters were uniformly adjusted over the entire image for better visualization. B-E) Higher magnification views of the same lobe stained with B) CCF2-AM, C) caveolin-1/Alexa Fluor 555 for identification of type I pneumocytes, D) pSP-C/Alexa Fluor 555 for identification of type II pneumocytes, and E) Gr1/Cy5 for identification of phagocytic cells. For panels B-E, scale bars represent 50 μm. (TIF) [file ppat.1004945.s001.tif]

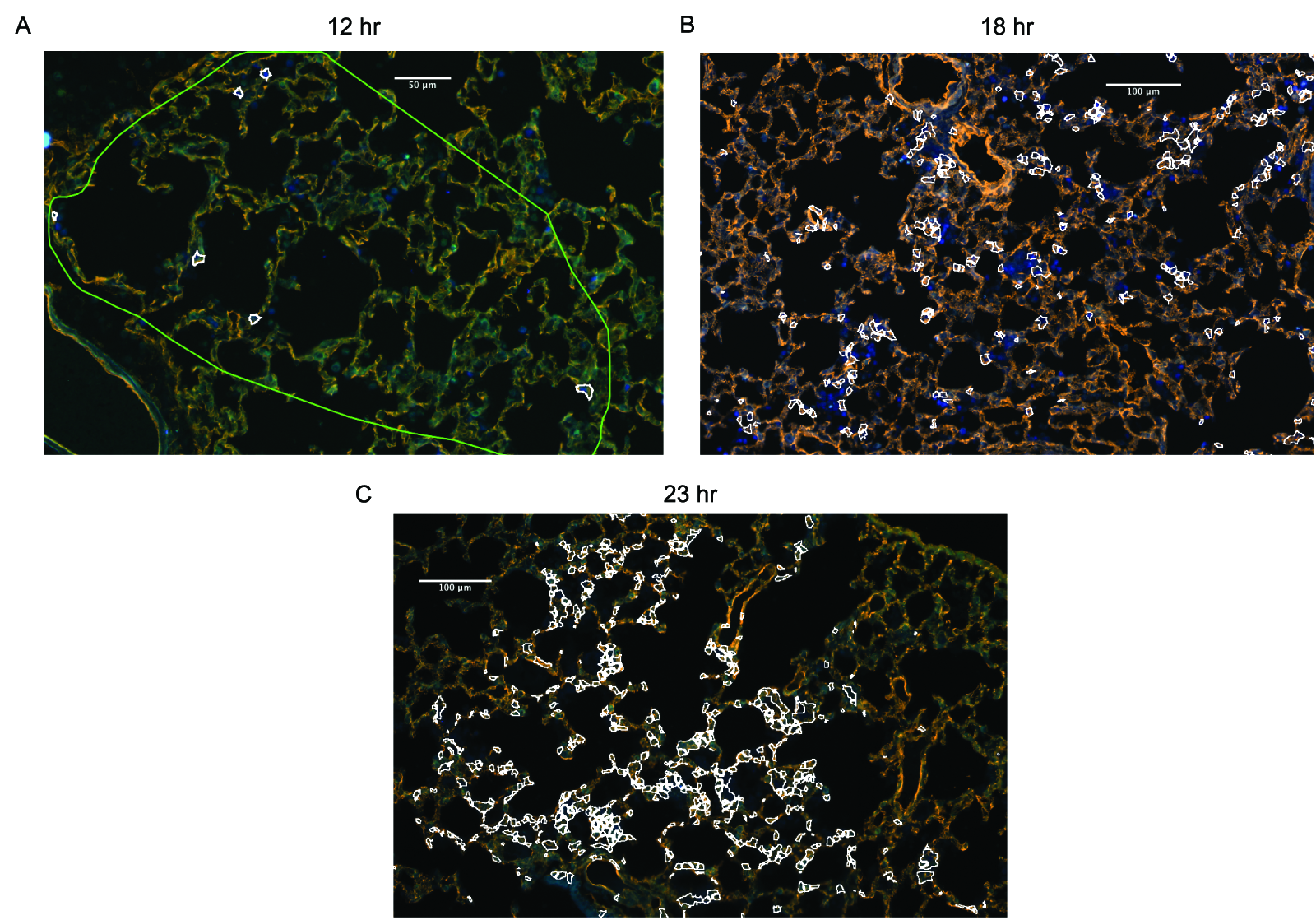

Supplement: S4 Fig — Each panel represents a FOCI and is taken from the white boxes shown in Fig 5. Type I pneumocytes (caveolin-1+ cells) are outlined in white. A) 12 hr post-infection. B) 18 hr post-infection. C) 23 hr post-infection. Scale bars represent 100 μm. (TIF) [file ppat.1004945.s004.tif]

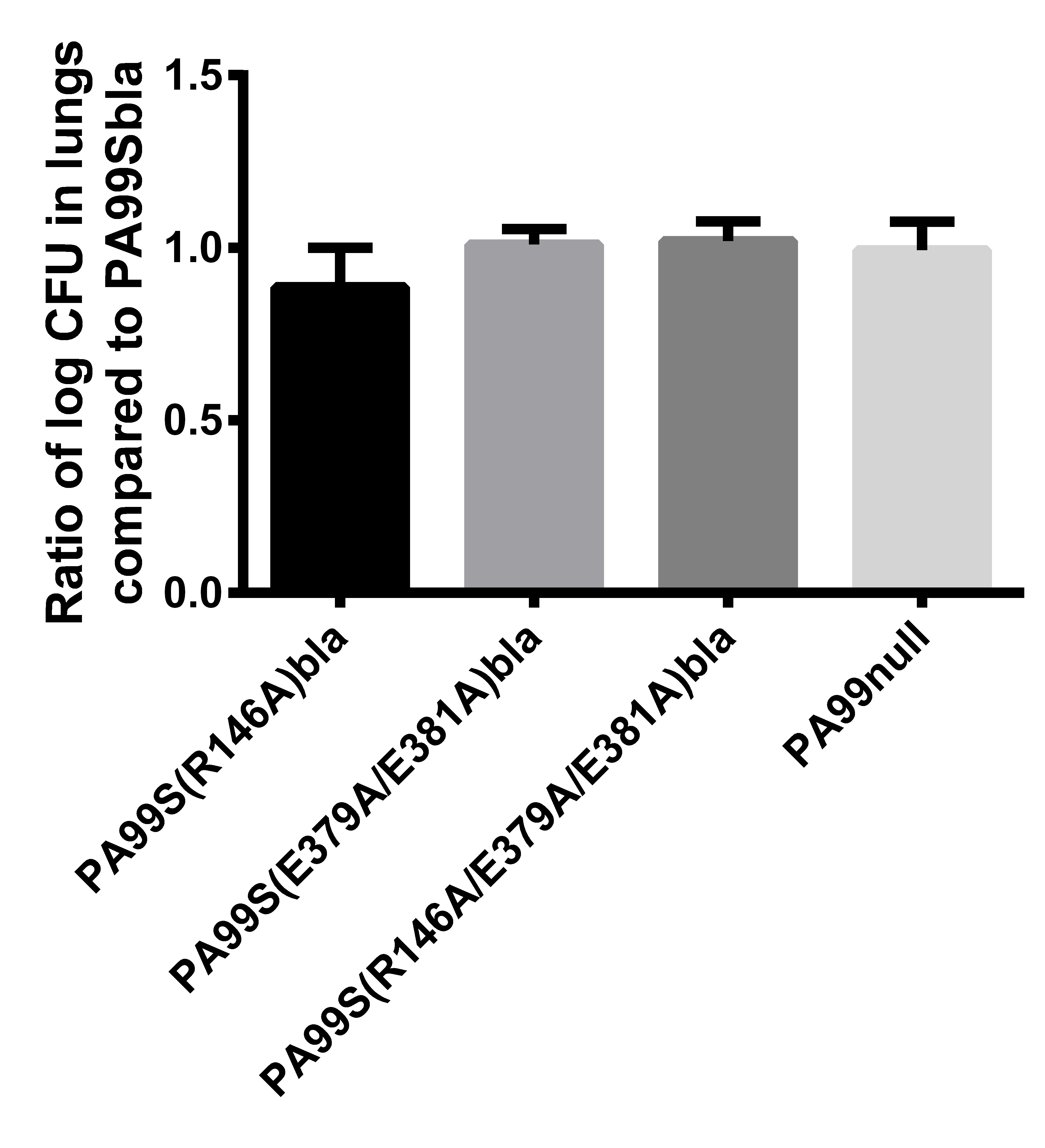

Supplement: S6 Fig — Mice were infected with 4.6 x 10^6^–9.2 x 10^6^ CFU PA99Sbla or PA99S(R146A)bla, 1.8 x 10^7^ CFU PA99S(E379A/E381)bla, 1.8 x 10^7^ CFU PA99S(R146A/E379A/E381A)bla, or 1.8 x 10^7^ PA99null bacteria. At 23 hr post-infection, lungs were removed, homogenized and plated. The average CFU of ExoS mutant strains recovered from whole lungs of mice were normalized to the number of CFU of PA99Sbla recovered at the same time point. Error bars represent SEM. n ≥ 3 per strain. (TIF) [file ppat.1004945.s006.tif]

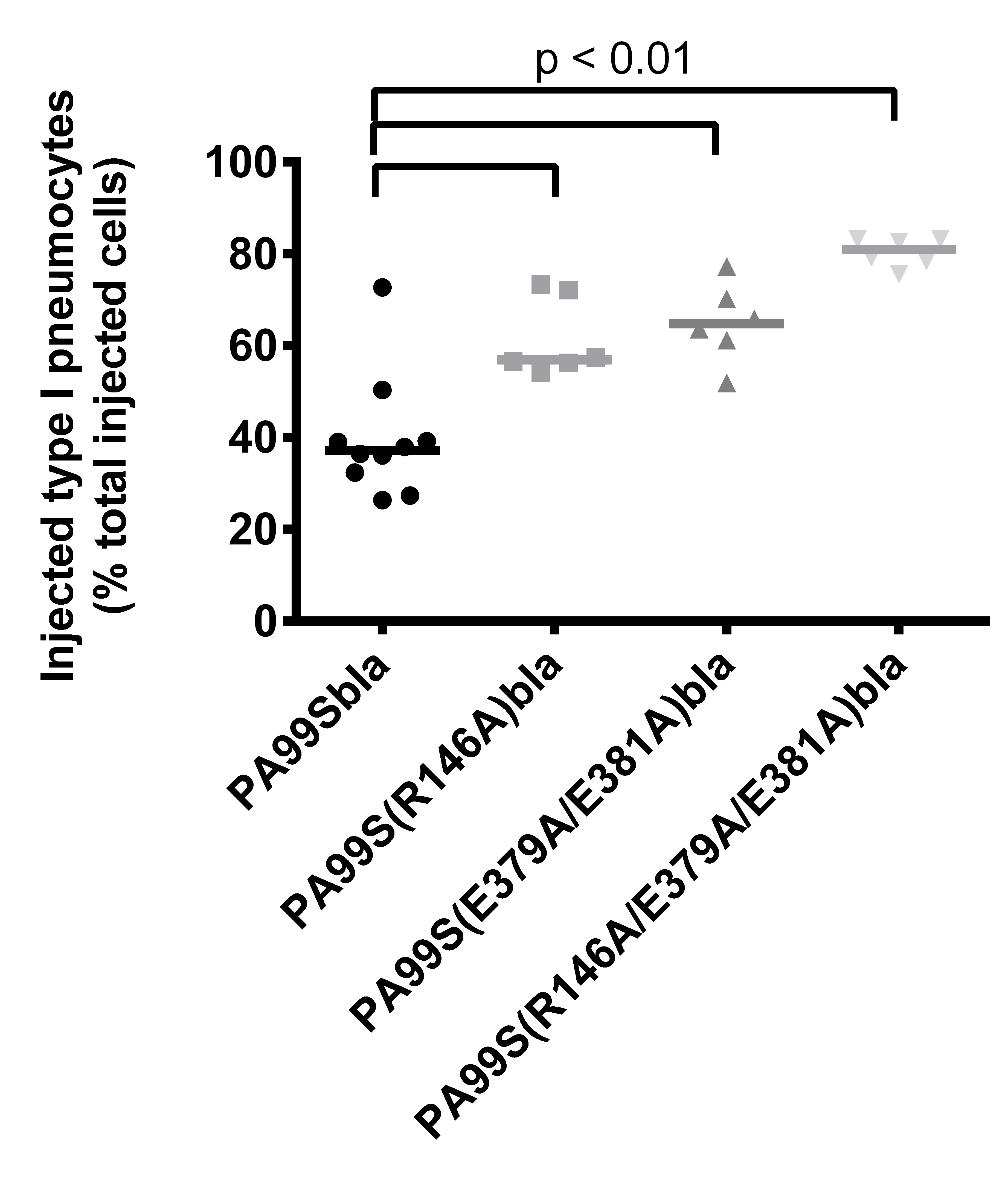

Supplement: S7 Fig — The proportion of injected cells that were type I pneumocytes varied with the enzymatically inactive form of ExoS secreted by the infecting bacteria. Lungs were harvested at 23 hr post-infection. Each symbol represents the value measured from a cross-section of an entire lung lobe. At least 6 lobes were analyzed per strain. Bars indicate medians. (TIF) [file ppat.1004945.s007.tif]
