# Supplementary material for: The Role of ExoS in Dissemination of Pseudomonas aeruginosa during Pneumonia
Source: PLoS Pathog. 2015 Jun 19;11(6):e1004945. doi: 10.1371/journal.ppat.1004945 (PMC4474835; doi:10.1371/journal.ppat.1004945)
Supplement: S1 Table — (DOCX) [file ppat.1004945.s010.docx]

| **Strains** | **Relevant characteristics** | **Source** |
| --- | --- | --- |
| **Bacterial strains** |  |  |
| **PA99** | **clinical isolate** | **[1, 2]** |
| **PA99null+S** | **PA99null complemented with *exoS* in the *attB* locus** | **[3]** |
| **PA99Sbla** | **PA99null complemented with *exoS-bla* in the *attB* locus** | **This study** |
| **PA99S(R146A)bla** | **PA99null complemented with *exoS(R146A)bla* in the *attB* locus** | **This study** |
| **PA99S(E379A/E381A)bla** | **PA99null complemented with *exoS(E379A/E381A)bla* in the *attB* locus** | **This study** |
| **PA99S(R146A/E379A/E381A)bla** | **PA99null complemented with *exoS(R146A/E379A/E381A)bla* in the *attB* locus** | **This study** |
| **PA99*ΔpscJ*** | **Secretes no effectors; does not make secretion apparatus** | **[3]** |
| **PA99*ΔpscJ*+Sbla** | **PA99*ΔpscJ* with *exoS-bla* in the *attB* locus** | **This study** |
| **PAK** | **Laboratory isolate** | **[4]** |
| **PAK+S(R146A/E379A/E381A)bla** | **PAK complemented with *exoS(R146A/E379A/E381A)bla* in the *attB* locus** | **This study** |
| **PAK*ΔS*** | **PAK*exoS*::omega** | **[5]** |
| **PAK*ΔS+* S(R146A/E379A/E381A)bla** | **PAK*ΔS* complemented with *exoS(R146A/E379A/E381A)bla* in the *attB* locus** | **This study** |
| **BL12** | **clinical bloodstream isolate** | **This study** |
| **BL12+ S(R146A/E379A/E381A)bla** | **BL12 complemented with *exoS(R146A/E379A/E381A)bla* in the *attB* locus** | **This study** |
| ***E. coli* S17.1** | **Used for conjugation** | **[6]{Simon, 1983 #260}** |
| **Plasmids** |  |  |
| **mini-CTX*exoS*** | ***exoS* gene and promoter ligated into mini-CTX-1; Tet^r^** | **[3] {Shaver, 2004 #1558}** |
| **mini-CTX*exoSbla*** | **mini-CTX*exoS* with 3’ *bla* fusion; Tet^r^** | **This study** |
| **mini-CTX*exoS(R146A)bla*** | **mini-CTX*exoS(R146A)* with 3’ *bla* fusion; Tet^r^** | **This study** |
| **mini-CTX*exoS*(*E379A/E381A)bla*** | **mini-CTX*exoS(E379A/E381A)* with 3’ *bla* fusion; Tet^r^** | **This study** |
| **mini-CTX*exoS(R146A/E379A/E381A)bla*** | **mini-CTX*exoS(R146A/E379A/E381A)* with 3’ *bla* fusion; Tet^r^** | **This study** |
| **pBR322** | **source of gene encoding the TEM-1 β-lactamase** | **[7]{Bolivar, 1977 #3793}** |
