# Supplementary material for: The Role of ExoS in Dissemination of Pseudomonas aeruginosa during Pneumonia
Source: PLoS Pathog. 2015 Jun 19;11(6):e1004945. doi: 10.1371/journal.ppat.1004945 (PMC4474835; doi:10.1371/journal.ppat.1004945)
Supplement: S1 File — (DOCX) [file ppat.1004945.s011.docx]

**SUPPLEMENTAL MATERIALS AND METHODS**

**Immunoblot analysis.** Bacteria were grown in 5 ml MINS medium at 37°C overnight with shaking. Culture supernatants were precipitated with ammonium sulfate (final concentration of 55%) for 3-24 hr on ice. Precipitated protein was recovered by centrifugation at 12,000 x *g* for 20 min at 4°C. The pellet was resuspended in 125 µl 10 mM NaCl, 125 µl 2X SDS-polyacrylamide gel electrophoresis sample buffer, and boiled for 5-10 min. A total of 10-40 µg of protein per sample was electrophoresed through a 10% (w/v) SDS-polyacrylamide gel. Proteins were transferred to a nitrocellulose membrane and probed with polyclonal rabbit antisera that recognized ExoS/ExoT [1]{Hauser, 1998 #448}. Goat anti-rabbit IRDye 680 conjugate (Li-COR, Lincoln, NE) was used as a secondary antibody. The membrane was imaged using a LiCOR Odyssey apparatus.

**Detection of ExoS-bla injection in vitro.** J774 macrophage-like cells were seeded into black 96-well tissue culture treated plates (PerkinElmer Inc., Waltham, MA) and allowed to adhere for one day. Cells were infected at an MOI of 10 for 3 hr and then loaded for 1 hr at room temperature with 1 mM CCF2-AM (Life Technologies, Carlsbad, CA). Fluorescence was quantified using a SpectraMax M3 fluorescence plate reader (Molecular Devices, Sunnyvale, CA). Excitation was set at 409 nm; emission was measured at 460 nm for blue fluorescence and 530 nm for green fluorescence. ExoS injection was determined by the ratio of blue to green fluorescence intensity as measured in relative fluorescence units (RFU): Injection = (RFU**_450nm_**_, sample_-RFU**_450nm_**_, background_)/(RFU**_520nm_**_, sample_ -RFU**_520nm_**_, background_), where background fluorescence was taken as fluorescence emissions from cells without CCF2-AM.

**SUPPLEMENTAL REFERENCES**

1. Feltman H, Schulert G, Khan S, Jain M, Peterson L, Hauser AR. Prevalence of type III secretion genes in clinical and environmental isolates of *Pseudomonas aeruginosa*. Microbiology. 2001;147:2659-69.

2. Ozer EA, Allen JP, Hauser AR. Characterization of the core and accessory genomes of *Pseudomonas aeruginosa* using bioinformatic tools Spine and AGEnt. BMC Genomics. 2014;15:737.

3. Shaver CM, Hauser AR. Relative contributions of *Pseudomonas aeruginosa* ExoU, ExoS, and ExoT to virulence in the lung. Infection and immunity. 2004;72:6969-77.

4. Bradley DE, Pitt TL. Pilus dependence of four *Pseudomonas aeruginosa* bacteriophages with non-contractile tails. J Gen Virol. 1974;24:1-15.

5. Kaufman MR, Jia J, Zeng L, Ha U, Chow M, Jin S. *Pseudomonas aeruginosa* mediated apoptosis requires the ADP-ribosylating activity of ExoS. Microbiology. 2000;146:2531-41.

6. Simon R, Priefer U, Puhler A. A broad host range mobilization system for in vivo genetic engineering: transposon mutagenesis in gram negative bacteria. Biotechnology. 1983;1:784-91.

7. Watson N. A new revision of the sequence of plasmid pBR322. Gene. 1988;70(2):399-403. Epub 1988/10/30. PubMed PMID: 3063608.

8. Hauser AR, Kang PJ, Engel J. PepA, a novel secreted protein of *Pseudomonas aeruginosa,* is necessary for cytotoxicity and virulence. Molecular microbiology. 1998;27:807-18.
